# Combined transcriptome and proteome profiling of the pancreatic β-cell response to palmitate unveils key pathways of β-cell lipotoxicity

**DOI:** 10.1186/s12864-020-07003-0

**Published:** 2020-08-26

**Authors:** Maria Lytrivi, Kassem Ghaddar, Miguel Lopes, Victoria Rosengren, Anthony Piron, Xiaoyan Yi, Henrik Johansson, Janne Lehtiö, Mariana Igoillo-Esteve, Daniel A. Cunha, Lorella Marselli, Piero Marchetti, Henrik Ortsäter, Decio L. Eizirik, Miriam Cnop

**Affiliations:** 1 ULB Center for Diabetes Research, Université Libre de Bruxelles, CP-618, Route de Lennik 808, 1070 Brussels, Belgium; 2 Division of Endocrinology, Erasmus Hospital, Université Libre de Bruxelles, Brussels, Belgium; 3 Diabetes Research Unit, Department of Clinical Science and Education, Sodersjukhuset, Karolinska Institutet, Stockholm, Sweden; 4 Clinical Proteomics Mass Spectrometry, Department of Oncology-Pathology, Karolinska Institutet, Science for Life Laboratory, 171 21 Solna, Sweden; 5 Department of Clinical and Experimental Medicine, University of Pisa, Pisa, Italy

**Keywords:** Pancreatic islets, Beta-cells, Free fatty acids, Lipid metabolism, Endoplasmic reticulum stress, RNA-sequencing, Proteome, Type 2 diabetes

## Abstract

**Background:**

Prolonged exposure to elevated free fatty acids induces β-cell failure (lipotoxicity) and contributes to the pathogenesis of type 2 diabetes. In vitro exposure of β-cells to the saturated free fatty acid palmitate is a valuable model of lipotoxicity, reproducing features of β-cell failure observed in type 2 diabetes. In order to map the β-cell response to lipotoxicity, we combined RNA-sequencing of palmitate-treated human islets with iTRAQ proteomics of insulin-secreting INS-1E cells following a time course exposure to palmitate.

**Results:**

Crossing transcriptome and proteome of palmitate-treated β-cells revealed 85 upregulated and 122 downregulated genes at both transcript and protein level. Pathway analysis identified lipid metabolism, oxidative stress, amino-acid metabolism and cell cycle pathways among the most enriched palmitate-modified pathways. Palmitate induced gene expression changes compatible with increased free fatty acid mitochondrial import and β-oxidation, decreased lipogenesis and modified cholesterol transport. Palmitate modified genes regulating endoplasmic reticulum (ER) function, ER-to-Golgi transport and ER stress pathways. Furthermore, palmitate modulated cAMP/protein kinase A (PKA) signaling, inhibiting expression of PKA anchoring proteins and downregulating the GLP-1 receptor. SLC7 family amino-acid transporters were upregulated in response to palmitate but this induction did not contribute to β-cell demise. To unravel critical mediators of lipotoxicity upstream of the palmitate-modified genes, we identified overrepresented transcription factor binding sites and performed network inference analysis. These identified LXR, PPARα, FOXO1 and BACH1 as key transcription factors orchestrating the metabolic and oxidative stress responses to palmitate.

**Conclusions:**

This is the first study to combine transcriptomic and sensitive time course proteomic profiling of palmitate-exposed β-cells. Our results provide comprehensive insight into gene and protein expression changes, corroborating and expanding beyond previous findings. The identification of critical drivers and pathways of the β-cell lipotoxic response points to novel therapeutic targets for type 2 diabetes.

## Background

The prevalence of type 2 diabetes has dramatically risen over the past few decades, reaching currently 9% of the world population [1]. This rise, expected to continue in the coming years, can be in large part explained by the global epidemic of obesity. Obesity increases the risk of the developing type 2 diabetes 7-fold [2] and is associated with increased circulating levels of free fatty acids (FFA), due to resistance to the anti-lipolytic effect of insulin and increased adipose tissue mass [3]. The increase in FFA may be a crucial link between obesity and the pathogenesis of diabetes. There is a substantial body of evidence supporting that elevated FFA have deleterious effects on β-cell function and survival, a phenomenon commonly referred to as lipotoxicity [4–6]. Prolonged elevation of FFA in obese [7] or genetically predisposed individuals [8] impairs glucose-stimulated insulin secretion. In animal models, high-fat feeding impairs the ability of β-cells to compensate for insulin resistance [9, 10] and induces β-cell apoptosis [11].

Palmitate is the most prevalent saturated FFA in humans. In large-scale epidemiological studies in the United States [12, 13] and Europe [14], high circulating levels of palmitic acid were associated with higher risk of type 2 diabetes, insulin resistance and inflammation [12]. Among different saturated FFA, palmitic acid had the strongest association with type 2 diabetes risk [14]. In vitro, palmitate is more toxic to β-cells than the unsaturated FFA oleate [15]. Prolonged exposure to palmitate decreases glucose-stimulated insulin secretion in rat [16] and human islets [17, 18] and induces β-cell apoptosis [15]. Even if exposure to a single FFA cannot fully reflect the complex metabolic environment to which β-cells are exposed in vivo [19], exposure to palmitate mimics pathophysiological changes relevant for β-cell failure observed in type 2 diabetes [6]. Thus, it is a useful model to explore mechanisms of lipotoxicity.

Omics studies leverage high-throughput technologies to decipher the intricate biological processes underlying complex diseases, such as diabetes. RNA-sequencing (RNA-seq) enables the interrogation of the whole transcriptome and has become the method of choice for transcriptome profiling. Compared to microarrays, RNA-seq has improved accuracy in transcript quantification, higher dynamic range and provides information on alternative splicing and novel transcripts [20]. As quantitative proteomics offered limited depth of coverage until recently, changes in transcript levels detected by transcriptome studies have been widely used as a surrogate for protein expression changes.

Compared to the separate analysis of transcript and protein changes induced by an environmental insult, the joint analysis of such datasets is a powerful tool to validate key expression changes and clarify whether transcript abundance mediates protein alterations. The integration of complementary layers of information generated by transcriptomics and proteomics is challenging, due to biological, technical and computational pitfalls [21]. However, it holds great potential for providing a more comprehensive view of gene expression regulation involved in human disease.

Our group has previously performed RNA-seq of human islets exposed to palmitate, as an in vitro model to better understand the response of β-cells to metabolic stress [22]. In the present study, we crossed this transcriptomic study (with the addition of one more experiment) with a proteome analysis of palmitate-treated rat INS-1E cells. We used both targeted and unbiased bioinformatic analyses to identify critical pathways and regulators of the β-cell response to lipotoxicity that are preserved among two different species, suggesting relevant functional impact. To elucidate the interactions between differentially expressed genes, we used the output of a random forests algorithm applied on expression data, together with literature information, to predict a gene regulatory network. Finally, we interfered experimentally with the expression of selected target genes to explore their role in β-cell function and survival.

## Results

### Transcript and protein profiling of pancreatic β-cells

Six human islet preparations exposed to palmitate for 48 h were profiled by RNA-seq. Five of these preparations (number 1–5 in Supplementary Table 1) have been previously published [22], while preparation 6 was new; RNA-seq of these 6 preparations was reanalyzed as described [22]. The donor characteristics are provided in Supplementary Table 1. RNA-seq identified 26,346 different gene transcripts, out of which 1087 were upregulated and 2333 downregulated. Proteomic profiling was performed in INS-1E cells following time course exposure to palmitate at 0, 4, 16 and 24 h (*n* = 2). This detected 7091 proteins, 394 of which were identified as outliers (as defined in the proteomics methods) and removed. 6333 proteins were successfully mapped to gene names and these genes/proteins were selected for further analysis. After this selection, the number of up- and down-regulated genes became 540 and 955 respectively. The number of up- and down-regulated proteins became 744 and 647 respectively. Comparison between RNA-seq and proteome revealed 85 upregulated and 122 downregulated genes common to both datasets (*p*-value for the intersection < 1 × 10^− 5^, from a hypergeometric distribution).

### Comparison of INS-1E cell and human islet gene expression profiles

**Fig. 1:**
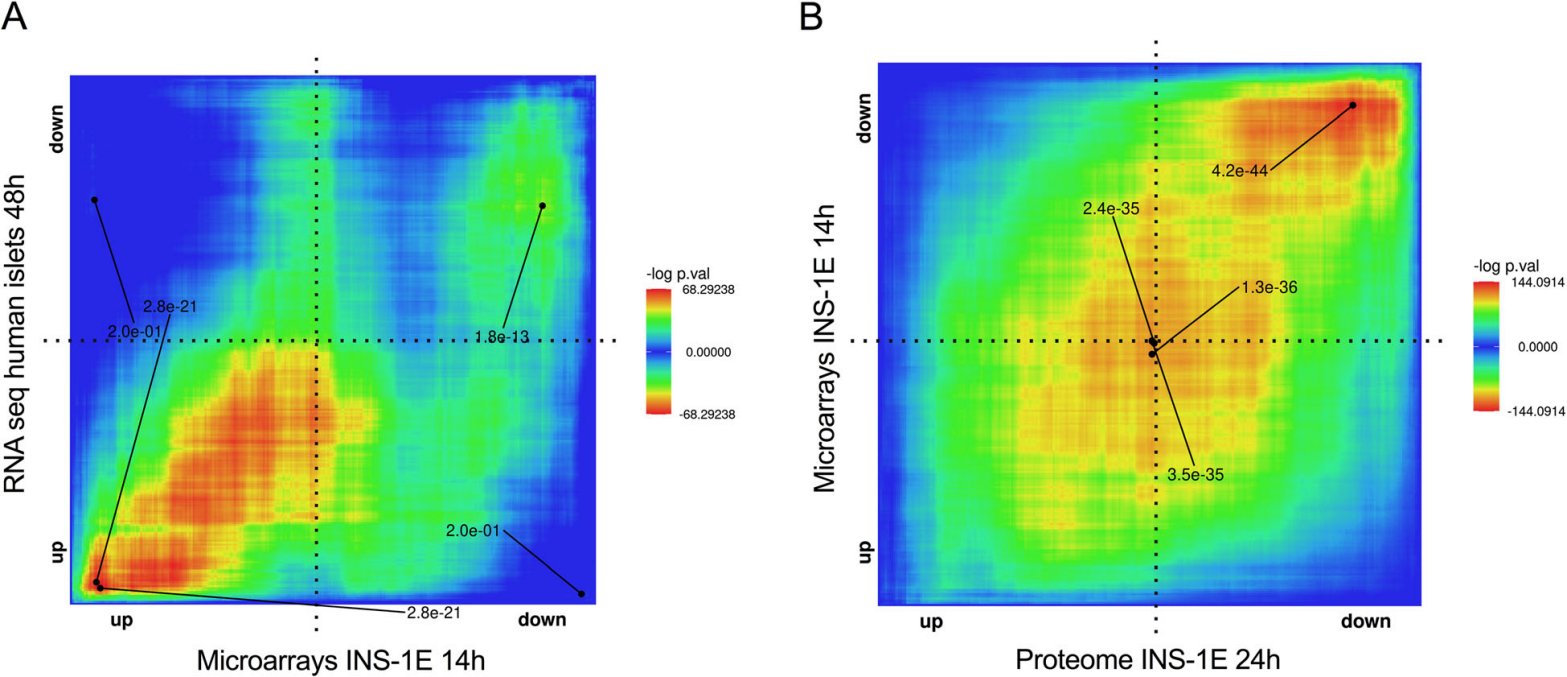
RRHO map showing highly significant overlap between human islet RNA-seq and INS-1E cell transcriptomes (**a**) and between microarrays and proteome of INS-1E cells following palmitate exposure (**b**). RNA-seq in human islets, microarrays in INS-1E cells and proteome in INS-1E cells were performed after exposure to palmitate for 48 h, 14 h and 24 h, respectively. Genes are ranked by fold change from most up- to most down-regulated. The level map colors represent -log *p*-values for overlap between ranked genes, with an indication of the smallest FDR corrected p-value for coordinates with the most statistically significant overlap between genes up-regulated in both datasets (bottom left corner) and down-regulated in both (top right corner)

In order to test the biological relevance of crossing omics data between species, we compared the human islet RNA-seq data with our previous INS-1E cell microarray data [23]. For this comparison, we used the rank-rank hypergeometric overlap (RRHO) method. RRHO treats data from two unrelated datasets as a ranked continuum of differential expression changes and searches for coordinated changes in gene expression in a threshold-free manner [24]. This robust method captures statistically significant and biologically relevant changes that may be missed when user-defined thresholds are applied [24]. RRHO heatmaps allow to visualize the pattern and significance of the overlap. Perfectly correlated gene expression changes in two datasets generate a strong positive signal along the diagonal in the RRHO heatmap (Supplementary Fig. 1A), whereas perfect overlap limited to the most up- and downregulated genes translates to a significant signal at the bottom left and upper right corners of the map, respectively (Supplementary Fig. 1B). When comparing two random gene sets, the heatmap shows absence of significant overlap throughout (Supplementary Fig. 1C). Comparing human islet RNA-seq data with INS-1E cell microarray data following palmitate exposure revealed highly significant overlap, most prominent for upregulated genes (Fig. 1a, bottom left corner) but also for downregulated genes (upper right corner). We further compared the INS-1E cell microarray profiling with the INS-1E cell proteome following palmitate exposure. The RRHO map showed highly significant overlap along the diagonal of differential expression (Fig. 1b), reminiscent of the map obtained when two independent datasets have identical expression changes (Supplementary Fig. 1A). These data indicate that gene signatures between rat INS-1E cells and human islets exposed to palmitate are significantly correlated, rendering their joint analysis in the present study pertinent.

### Analysis of palmitate-modified genes/proteins

**Fig. 2:**
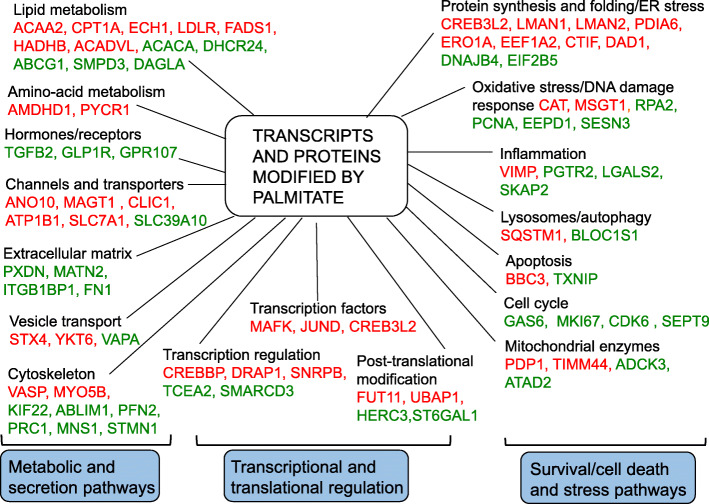
Manual classification of palmitate-modified β-cell transcripts and proteins into functional categories. Upregulated genes are shown in red and downregulated genes in green. The list of genes shown in this figure is not exhaustive

In a first step, we analyzed the 207 genes differentially expressed in both the RNA-seq and proteome by manually curating them into functional categories based on their presumed biological function (Fig. 2 and Supplementary Table 2). Exposure to palmitate produced a complex transcriptional and translational response. Among the functions that were most affected by palmitate were: lipid metabolism, channels and transporters, cytoskeleton and extracellular matrix. Additionally, palmitate caused gene expression changes related to cell cycle control, apoptosis and stress pathways, including endoplasmic reticulum (ER) stress and oxidative stress.

#### Lipid metabolism

Palmitate modulated the expression of genes with key roles in FFA metabolism. It induced expression of CPT1, which mediates mitochondrial FFA uptake, and enzymes involved in FFA β-oxidation, namely ACADVL, ACAA2 and HADHB. Palmitate inhibited expression of ACACA, an enzyme catalyzing fatty acid synthesis and lipogenesis, and induced FADS1, a desaturase that catalyzes the synthesis of long-chain omega-3 and omega-6 polyunsaturated fatty acids.

Moreover, palmitate modulated expression of genes involved in cholesterol biosynthesis and transport. Palmitate downregulated DHCR24, which catalyzes the final step in cholesterol biosynthesis, induced expression of the LDL receptor and attenuated ABCG1 expression. The latter acts as cholesterol efflux channel in other cell types. In β-cells, it affects the cholesterol content of insulin granules and granule morphology. Interestingly, its deficiency attenuates insulin secretion in mouse β cells [25].

#### ER stress

In previous studies by us and others, chronic exposure to palmitate has been shown to activate signaling through the three canonical branches of the ER stress response under the control of PERK, IRE1 and ATF6, and to elicit ER stress-induced apoptosis [15, 26].

CREB3L2 and other members of the CREB3/ATF family of transcription factors have been proposed as novel ER stress transducers, functioning in a cell- and stimulus-specific manner [27]. These ER membrane-bound proteins are activated by regulated intramembrane proteolysis at the Golgi in response to ER stress. CREB3L2 was upregulated by palmitate at the mRNA and protein level. This was confirmed by qPCR in INS-1E cells and independent human islet preparations (Supplementary Fig. 2A-B). Based on data suggesting a role of CREB3L2 against ER stress-induced apoptosis in other cell types [28, 29], we investigated whether CREB3L2 protects β-cells from lipotoxicity. CREB3L2 knockdown by siRNA in human islet cells did not alter palmitate-induced apoptosis (Supplementary Fig. 2C-D). Interestingly, CREB3L2 silencing by two siRNAs targeting different mRNA regions in INS-1E cells decreased glucose-stimulated insulin secretion, without affecting insulin content (Supplementary Fig. 2E-G). These data unveil a role of CREB3L2 in regulating insulin secretion, and not in lipoapoptosis.

Palmitate induced LMAN1 and LMAN2 expression. LMAN1 is known to act as a cargo receptor for selective glycoprotein transport between the ER and Golgi compartments and the early secretory pathway [30]. Palmitate has been shown to inhibit protein trafficking between ER and the Golgi, inducing ER stress by subsequent protein overload in the ER [31]. Palmitate also upregulated ERO1A, an oxidoreductase involved in oxidative protein folding in the ER. The palmitate-induced ERO1A upregulation may be deleterious, since ERO1A hyperactivity has been reported to lead to oxidative perturbations, eliciting ER stress [32]. Finally, palmitate downregulated EIF2B5, which codes for the catalytic eIF2Bε subunit of the eukaryotic initiation of translation factor eIF2B [33]. The latter is a regulator of protein translation by exchanging GDP for GTP in eIF2, enabling the ternary complex to form and translation to initiate. In conditions of ER stress and PERK-dependent eIF2α phosphorylation, eIF2B function is inhibited; our current findings also suggest transcriptional and translational regulation of the protein.

#### Channels and transporters

Palmitate modified the expression of several channels and transporters. It upregulated CLIC1, an intracellular chloride ion channel, previously shown to act as a downstream effector of insulin [34], and it enhanced expression of members of the SLC7 family of amino-acid transporters. SLC7A1 was upregulated at both protein and mRNA level and SLC7A5 and SLC7A11 were upregulated at the mRNA level. It has been previously shown that SLC7A1 and SLC7A5, as well as other amino-acid transporters, are induced during ER stress in β-cells. This is part of an anabolic program to overcome translational repression secondary to PERK-dependent eIF2α phosphorylation [35] and it was suggested to contribute to β-cell demise. To test this hypothesis, we evaluated the effect of SLC7A1 depletion in β-cells under lipotoxic conditions and following exposure to the chemical ER stressor cyclopiazonic acid (CPA). SLC7A1 knockdown in INS-1E cells induced apoptosis under basal conditions and increased palmitate and CPA toxicity (Supplementary Fig. 3A-D). Similarly, SLC7A5 inhibition by RNA interference (Supplementary Fig. 3E-F) or by the chemical inhibitor BCH (Supplementary Fig. 3G) did not protect from palmitate-induced apoptosis. Thus, the upregulation of these amino-acid transporters by lipotoxic ER stress does not mediate cell death.

#### Hormones and receptors

Palmitate inhibited expression of the GLP1 receptor in our –omic studies. This is in keeping with previous reports demonstrating that palmitate downregulates the GLP1 receptor, which decreases the β-cell insulin secretory response and impairs the effect of GLP1 agonists [36].

### Pathway analysis and enriched transcription factors

**Fig. 3:**
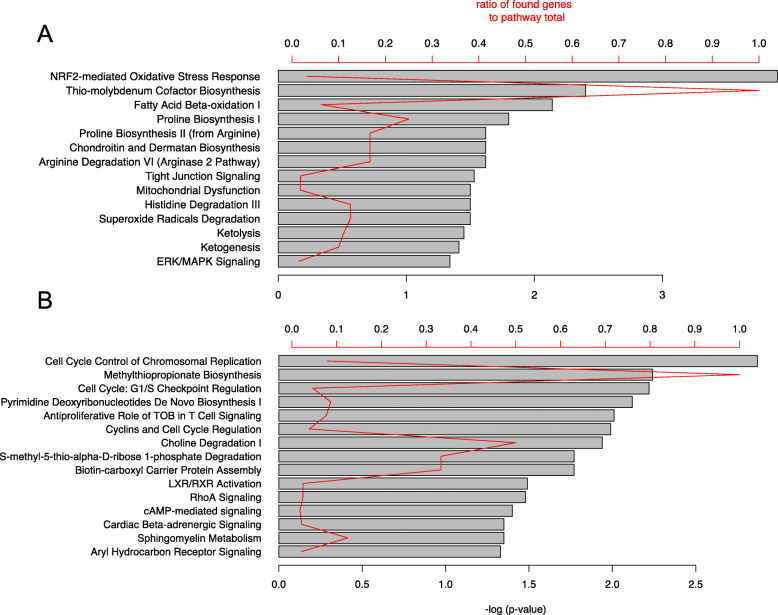
Enriched pathways in differentially expressed genes/proteins in palmitate-treated β-cells. Upregulated (**a**) and downregulated genes (**b**) were analyzed separately by IPA. The length of the bars is proportional to the significance of the association between the set of genes and the pathway, expressed by the negative logarithm of the p-value. The red line indicates the ratio of modified genes/proteins mapping to the pathway to the total number of elements of this pathway. Only pathways with *p* < 0.05 are shown

Further to our manual annotation, we used bioinformatic approaches to obtain an unbiased overview of the biological pathways modified in lipotoxic conditions. Genes modified by palmitate at both mRNA and protein level were analyzed by Ingenuity Pathway Analysis (IPA, QIAGEN Inc., https://www.qiagenbioinformatics.com/products/ingenuity-pathway-analysis) to identify enriched pathways. IPA evaluates the overrepresentation of a group of genes mapping to a specific pathway compared to a reference set of genes and calculates significance for each pathway of interest. IPA indicated that upregulated genes were involved principally in the oxidative stress response, fatty acid β-oxidation, mitochondrial dysfunction, the ERK/MAPK pathway and amino-acid metabolism (Fig. 3a). Downregulated genes were overrepresented in cell cycle regulating pathways, LXR/RXR signaling and cAMP-mediated signaling (Fig. 3b).

**Fig. 4:**
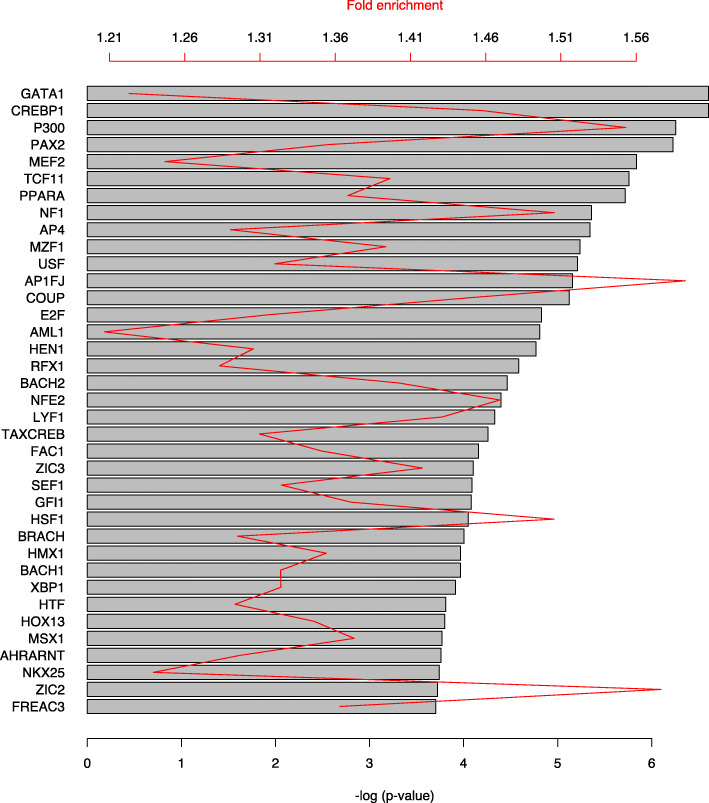
Enriched transcription factors in palmitate-modified genes/proteins in β-cells. Genes differentially expressed at both mRNA and protein level were analyzed by DAVID (UCSC_TFBS). The length of the bars is proportional to the significance of the overrepresentation of potential binding sites for the indicated transcription factors in the modified genes, expressed by the negative logarithm of the p-value. The red line indicates the fold enrichment of the palmitate-modified genes compared to a random set of genes from the human genome

In order to identify transcription factors orchestrating the palmitate-induced gene expression changes, the Database for Annotation, Visualization and Integrated Discovery (DAVID) was used to assess overrepresentation of transcription factors binding sites [37, 38]. Among the most enriched transcription factors were CREBP1, p300, PPARA, XBP1, BACH1 and BACH2 (Fig. 4).

### Network inference analysis

**Fig. 5:**
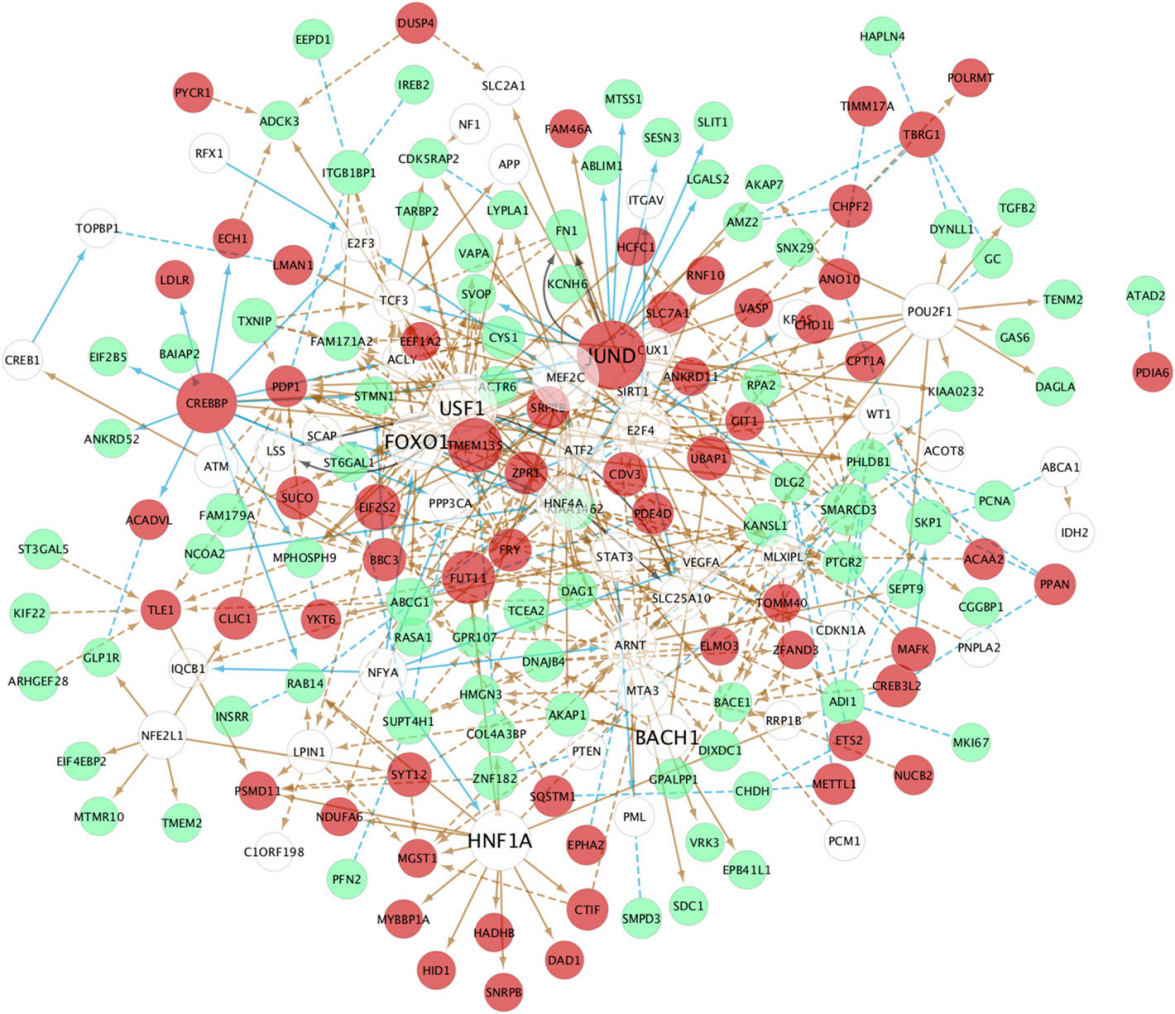
Network inference analysis of palmitate-modified genes/proteins. Enriched regulators identified by IPA and DAVID were added to the 207 differentially expressed genes, resulting in a dataset of 258 genes considered for this analysis. Upregulated genes are shown in red, downregulated genes in green, and the added regulators are shown in white circles. Regulatory networks were inferred in the RNA-seq and proteomics data separately using a random forest algorithm to score predictors and then the 2 networks were combined. Blue lines denote regulations identified in RNA-seq, yellow lines denote regulations identified in proteomics and black lines denote regulations found in both datasets. Regulations present in biological databases (IPA, DAVID, TRANSFAC, RegNetwork) are shown in continuous lines and regulations that have not been previously described are shown in dashed lines

Aiming to unravel potential key mediators of lipotoxicity, we combined the output of a random forests algorithm to infer regulations among the differentially expressed genes and proteins, and a prior network obtained from literature data (see Methods - Network inference analysis). To obtain this prior network, we searched IPA and DAVID for putative upstream regulators in the set of 207 differentially expressed genes/proteins. The obtained regulators (53 in total) were added to the set of 207 genes/proteins, resulting in a set of 258 genes/proteins (2 were already present). As shown in Fig. 5, we obtained a network of 416 regulations involving 190 genes/proteins: 3 regulations inferred from the RNA-seq and proteomics dataset, all present in the prior network; 97 inferred from the RNA-seq dataset, of which 44 were present in the prior network; and 316 inferred from the proteomics dataset, of which 129 were present in the prior network. The most significant hub genes with the highest number of interactions with palmitate-modified genes were USF1, MEF2C, JUND, HNF1A, FOXO1 and BACH1.

USF1 polymorphisms have been associated with increased risk for type 2 diabetes and metabolic syndrome [39–41]. The role of HNF1A and FOXO1 in β-cell pathophysiology is well established. HNF1A mutations cause maturity-onset diabetes of the young (MODY) type 3 and rare variants in this gene increase type 2 diabetes risk [42]. FOXO1 is a transcription factor acting as a key nutrient sensor, with a critical role in β-cell function and survival [43, 44].

BACH1 (BTB and CNC homology 1) is a transcription factor that binds to Maf-recognition elements (MAREs) and inhibits transcription of oxidative stress-responsive genes, including heme oxygenase-1 (HMOX1) [45]. qPCR showed a trend for BACH1 induction in INS-1E cells and significant upregulation of BACH1 in human islets exposed to palmitate (Supplementary Fig. 4A-B). BACH1 silencing in human islet cells did not protect from palmitate-induced apoptosis (Supplementary Fig. 4C-D). BACH1 knockdown in INS-1E cells, confirmed by qPCR of BACH1 and its target Hmox1 (Supplementary Fig. 4E-F), did not alter insulin content but it enhanced glucose- and potassium-stimulated insulin secretion (Supplementary Fig. 4G-I). Collectively, these findings suggest that BACH1 may contribute to lipotoxic β cell dysfunction.

## Discussion

The present study is the first to integrate transcriptomic and proteomic profiling of β-cells exposed to palmitate, to thoroughly map the lipotoxic response. Our group has previously published a transcriptome analysis of palmitate-treated human islets using RNA-seq [22]. This pointed to important pathways for lipotoxic β-cell demise, such as the ER stress response, suppression of autophagic flux and inhibition of transcription factors that control β-cell identity. This study, as well as microarray studies of human islets [18, 46] and clonal β-cells [47, 48] by other groups, have helped to expand our understanding of the underlying mechanisms of lipotoxicity. A limitation of transcriptomic studies is that transcriptional changes are not necessarily mirrored by protein expression [49–51]. This can be explained by post-transcriptional regulation that affects mRNA stability and translation rate (microRNAs, RNA binding proteins), post-translational modifications, and protein turnover. As an example, palmitate has been shown to alter the expression of non-coding RNAs [52] and the lysine acetylation of proteins [53]. Therefore, crossing transcriptomic with proteomic data provides valuable insights that may not be uncovered by the individual analysis of mRNA and protein expression.

In the present study, we used state-of-the art, highly sensitive transcriptome and proteome profiling technologies. These allowed deep coverage of the transcriptome and detection of least 2–3 times more proteins compared to previous proteomic studies of palmitate-treated β-cells [17, 54–56]. We focused our analyses on the subset of differentially expressed genes that were common to the RNA-seq and proteomic datasets, i.e. changes in transcript abundance resulted in protein expression changes, strengthening the validity and functional relevance. The targeted analysis of our data, together with the unbiased, bioinfomatic analyses, unveiled pathways and transcription factors with important roles for the palmitate-induced β-cell dysfunction and death.

### Lipid metabolism

In accordance with previous microarray [46, 48] and proteomic studies [55], palmitate induced extensive changes in genes involved in lipid metabolism. Palmitate triggered changes consistent with increased FFA β-oxidation, reduced lipogenesis and modified cholesterol metabolism. CPT1, the rate-limiting enzyme in mitochondrial FFA import, was upregulated and ACACA, a key enzyme driving lipogenesis, was downregulated. Interestingly, it has been shown that adenoviral CPT1 overexpression in β-cells impairs glucose-stimulated insulin secretion [57]. A recent study found that short-term inhibition of ACACA in mice impaired insulin secretion and long-term ACACA inhibition decreased β-cell mass [58]. The palmitate-induced lipid metabolism gene/protein expression changes identified in the present study may hence contribute to inhibit insulin secretion and affect β-cell mass. Our in silico analyses point to specific transcription factors orchestrating these palmitate-induced changes. Pathway analysis showed that palmitate modified LXR/RXR signaling. LXR is a transcription factor that forms heterodimers with RXR and regulates FFA β-oxidation, lipid synthesis and storage. LXR agonists have been shown to modulate palmitate toxicity in β-cells [59, 60]. Transcription factor enrichment analysis by DAVID identified PPARα as a critical player in the transcriptional response. PPARα is predicted in silico to bind to the large majority of the palmitate-modulated genes related to lipid metabolism.

Our network inference analysis indicated FOXO1 as a major regulator of the β-cell response to palmitate. FOXO1 signaling was identified as an enriched pathway in a previous proteomic study of palmitate-exposed human islets [17]. FOXO1 is a multifunctional protein regulating proliferation, differentiation, apoptosis and metabolic pathways in β-cells [44]. FOXO1 inhibition in vitro protects β-cells from FFA-induced apoptosis [61]. On the other hand, ablation of FOXO1 in mice increases palmitate oxidation in a PPARα-dependent way and impairs glucose-stimulated insulin release [43]. FOXO1-deficient cells have impaired ability to switch from lipid to glucose oxidation, becoming metabolically inflexible [43], which can ultimately lead to loss of β-cell identity and dedifferentiation [62]. Taken together, our data suggest that LXR, PPARα and FOXO1 are key transcription factors for the metabolic adaptation or dysfunction of β-cells in response to palmitate.

### cAMP/PKA signaling

A further finding of our pathway analysis was that palmitate modified cAMP/PKA signaling. The activation of PKA by cAMP is a cellular signaling pathway that regulates a variety of cellular responses. In keeping with the role of cAMP/PKA signaling in lipotoxicity, it has been shown that prolonged exposure to palmitate suppresses the glucose-induced cAMP increase and contributes to secretory defects [63]. Raising intracellular cAMP levels confers protection against palmitate-induced apoptosis [64].

Compartmentalization of PKA is essential to elicit distinct cellular effects. This is mediated by the family of PKA anchoring proteins (AKAPs), which target PKA to discrete subcellular locations and integrate signals derived from multiple pathways [65]. Interestingly, palmitate downregulated 3 members of this family, namely AKAP1, AKAP7 and AKAP11. Palmitate also upregulated PDE4D, a phosphodiesterase that inactivates cAMP signaling, and VASP, a known target of PKA modifying actin cytoskeleton [66].

Activation of cAMP signaling mediates, at least in part, the insulinotropic and anti-apoptotic effects exerted by GLP1 [67, 68]. In this study, we confirm that palmitate represses GLP1 receptor expression, potentially contributing to decreased cAMP signaling.

### Oxidative stress

Palmitate also upregulated genes involved in oxidative stress pathways. FFA are important inducers of reactive oxygen species (ROS) through mitochondrial and non-mitochondrial pathways [69]. While short-term elevation of ROS stimulates insulin secretion, sustained or excessive ROS production inhibits insulin secretion [69].

Both in the DAVID and network inference analyses, BACH1 was predicted to be a key transcription factor mediating the response to palmitate. BACH1 is a crucial player in regulating antioxidant gene transcription, acting as a repressor of heme oxygenase-1 (HMOX1) and other antioxidant enzymes [70]. In mouse models, BACH1 deficiency confers protection against oxidative stress-induced diabetes [71]. In our functional studies, BACH1 inhibition did not affect apoptosis, but it enhanced insulin secretion and induced heme oxygenase-1. This points to a role of BACH1 in regulating insulin secretion, through the modulation of the oxidative stress response.

### Amino-acid metabolism and transport

Pathway analysis indicated that palmitate induced changes in amino-acid metabolism, in particular of proline, histidine and arginine. These were associated with the differential expression of two enzymes, PYCR1 that synthesizes proline from arginine and AMDHD, involved in histidine degradation. Furthermore, palmitate induced SLC7A1 expression, which transports the cationic amino-acids arginine, lysine and ornithine. In contrast to a previous report [35], we found that SLC7A1 and SLC7A5 silencing did not protect from lipotoxic or ER stress-induced apoptosis. Hence, the upregulation of amino-acid transporters during ER stress does not contribute to β-cell demise. Recent plasma metabolomic studies have shown a positive association between specific branched-chain amino-acids and type 2 diabetes [72]. Metabolomic studies of β-cells, basally and in the face of metabolic stress, will shed further light on the biological impact of amino-acid alterations.

### Cell cycle

In keeping with microarrays in INS-1E cells [48] and proteomics in human islets [17, 46], we found that cell cycle pathways were downregulated. FFA suppress glucose-stimulated β-cell proliferation in in vitro and in vivo rodent models [73]. Human β-cells express cell cycle regulating key molecules but, contrary to rodent cells, these molecules are refractory to activation by proliferative signals [74]. Given that adult human β-cells are post-mitotic and do not proliferate [75, 76], the downregulation of cell cycle regulating genes by palmitate is an intriguing finding. It may reflect gene expression changes in non-β-cells in human islets.

We acknowledge that this study has certain limitations. First, transcriptome was performed in human islets, while proteome was performed in clonal rat INS-1E β-cells, because of the amount of material needed for protein profiling. There are differences between rat and human β-cells, with respect to glucose sensing, redox regulation and susceptibility to cytotoxic agents [77]. Nevertheless, the comparison by RRHO of human islet and INS-1E cell transcriptomes shows that there is strong overlap between their palmitate-induced gene signatures (Fig. 1a). A previous quantitative comparison of human and rat β-cells demonstrated that the core proteomic architecture of β-cells is highly conserved. In particular, the relative abundance of glycolytic enzymes, Krebs cycle enzymes, β-oxidation enzymes, and oxidative phosphorylation are quite similar [78]. The comparison of gene/protein expression profiles between INS-1E cells and human islets is therefore relevant. Second, RNA-seq was performed in human islets, which is a mixed cell population containing 50% β-cells. It has been shown, however, that there is a high correlation between β-cell and islet-expressed genes (*r* = 0.94) and that 87% of the variance in β-cell gene expression can be explained using islet expression as a proxy [79]. Studies in human islets also suffer from variability, due to islet isolation procedures, clinical donor characteristics and (epi) genetic variation [80]. They are, nevertheless, the β-cell model closest to human (patho)physiology. Transcript and protein changes were assessed at different times in human islets and INS-1E cells. These time points were selected on the basis of previous functional studies, indicating that human islets are less sensitive than INS-1E cells to palmitate [15]. Finally, several studies have shown that the abundance of transcripts with detectable proteins in proteomics is shifted to higher expression values. This suggests that some transcripts do not yield functional proteins or yield lowly expressed proteins that are below the proteomics detection limit. Taking into account that RNA-seq is more sensitive in detecting quantitative changes in gene expression, we applied less strict criteria in the definition for differential expression from the proteomics database.

Notwithstanding these limitations, this is the first study to combine transcriptomic analyses with sensitive time course proteomic profiling of palmitate-exposed β-cells.

## Conclusions

This combined transcriptomic and proteomic study of β-cells provides a comprehensive overview of the gene expression changes induced by palmitate and highlights significant pathways implicated in the response to lipotoxicity. These include alterations in lipid metabolism, oxidative stress, ER stress, cAMP/PKA signaling and cell cycle pathways. Our bioinformatic analyses unveil transcription factors that may act as drivers of the response to FFA and could serve as targets for future investigations.

## Methods

### Human islets and rodent β-cells

Human islets were obtained from heart-beating organ donors with no medical history of diabetes or metabolic disorders. Islets were isolated using collagenase digestion and density gradient purification and cultured in M199 medium (at 5.5 mM glucose). The islets were shipped from Pisa to Brussels within 1–5 days of isolation. In Brussels, the human islets were cultured in Ham’s F-10 medium (at 6.1 mM glucose), containing 10% heat-inactivated FBS, 2 mM GlutaMAX, 50 mM 3-isobutyl-1-methylxanthine, 1% charcoal-absorbed BSA, 50 units/mL penicillin, and 50 mg/mL streptomycin. The islets were treated with 0.5 mM palmitate (Sigma, Schnelldorf, Germany) or control (ethanol) in the same medium containing 1% charcoal-absorbed BSA but no FBS for 48 h. The use of albumin to complex FFA is essential to ensure that the experiment is biologically relevant [5, 81]. Palmitate was dissolved in 90% ethanol, heated to 60 °C, and diluted 1:100 (final concentration 0.5 mM). The average percentage of β-cells in the human islet preparations, examined by insulin immunofluorescence [15], was 50%.

The rat insulinoma cell line INS-1E [82] (a kind gift from Professor Claes Wollheim, Centre Medical Universitaire, Geneva, Switzerland) was used between passages 61–72 and maintained in RPMI 1640 medium (SVA, Uppsala, Sweden) containing 11.1 mM glucose supplemented with 10% FBS (Invitrogen, Carlsbad, CA), 10 mM HEPES (Invitrogen), 2 mM L-glutamine (SVA), 1 mM sodium pyruvate (Sigma-Aldrich, St. Louis, MO), 50 μM β-mercaptoethanol (Sigma-Aldrich) and antibiotics (6 mg/ml penicillin G and 5 mg/ml streptomycin sulfate [Invitrogen]). INS-1E cells tested negative for Mycoplasma infection. 72 h prior to treatment cells were seeded in 100 mm Petri dishes at a density of 2,400,000 cells per dish in 15 ml medium. During palmitate exposure (0.4 mM, for 0, 4, 16 and 24 h), cells were kept in similar medium, but with a 1% FBS concentration and 0.5% FFA-free BSA (Roche Diagnostics GmbH, Mannheim, Germany). Untreated cells received equal amounts of ethanol and BSA. Two independent replicates were performed for each treatment. Palmitate was prepared in 12.5% ethanol as 100 mM stock solution, and, prior to treatment, complexed with BSA for 30 min at 37 °C.

### RNA-seq

RNA-seq was performed in 6 human islet preparations exposed to palmitate for 48 h or ethanol (control) and analyzed as previously described [22]. In order to assess differential expression, the exact Fisher test was applied on RNA-seq counts for each paired sample, followed by the Benjamini-Hochberg correction for multiple testing. Genes were considered as differentially expressed if they were significantly modified (*p* < 0.05) in the same direction in at least 4 out the 6 paired samples, and significantly modified in the opposite direction in at most 1 paired sample.

### Proteomics

Proteomics were performed on INS-1E cells following a time-course palmitate exposure at 0, 4, 16 and 24 h using an iTRAQ technique in 2 independent experiments. iTRAQ is based on the covalent labeling of the N-terminus and side chain amines of peptides from protein digestions with tags of varying mass. Samples were pooled and fractionated by liquid chromatography and analyzed by tandem mass spectrometry (MS/MS). A database search was performed using the fragmentation data to identify the labeled peptides and corresponding proteins. The fragmentation of the attached tag generates a low molecular mass reporter ion that was used to relatively quantify the peptides and proteins from which they originated [83]. A detailed description of the method is provided in the additional material (Supplementary Methods).

Protein expression was first divided by the expression mean of the control samples (i.e. the 2 samples at 0 h) of all proteins. Then, the expression of each protein at further time points was divided by the mean of its control samples. A 95% confidence interval for the ratio between the control samples was estimated (2 to the power of ±1.96*standard deviation of the log2 transformed ratio, assuming normality) and used as cut-off values for up and down-regulation (1.24 and 0.8 respectively). Proteins were considered outliers and excluded from analyses if the ratio of the control samples exceeded the estimated 95% confidence interval. A protein was considered differentially expressed if its expression was higher/lower than these cut-offs in any of the time points of exposure in at least one experiment. Rat proteins were mapped to human genes using Uniprot conversion tools (www.uniprot.org/) and NCBI’s homology database (https://www.ncbi.nlm.nih.gov/homologene).

### Rank-rank hypergeometric overlap (RRHO)

Rank-rank hypergeometric overlap (RRHO) maps were generated by a modified two-tailed RRHO method [24, 84]. Differentially expressed genes were ranked by the logarithm of fold change. The *p*-values of the overlapping genes were assessed by a two-tailed hypergeometric test and false discovery rate corrected by the Benjamini and Yekutieli method. The RRHO R package was modified to better take into account the multiplicity of minimal *p-*values, null *p-*values, the up- and down-regulated genes going in opposite direction and the asymmetry between the number of genes up- or down-regulated in the two datasets.

### Network inference analysis

Gene regulatory networks were obtained by combining inferred networks from the expression profiles of the proteomics and RNA-seq datasets and a prior network, obtained from literature knowledge.

#### Prior network

To obtain a prior network using literature data, we searched for putative upstream regulators of the set of 207 differentially expressed genes/proteins using IPA (QIAGEN, Redwood City) and DAVID. In DAVID, we looked for enriched transcription factor binding sites (source UCSC TFBS, selection criteria Benjamini-Hochberg adjusted *p* < 0.05). For IPA, the *upstream analysis* was used (criteria for selection non-adjusted *p* < 0.001). 53 regulators were obtained and added to the set of differentially expressed genes/proteins (2 of them were already present - the added 51 regulators are ATF2, MEF2C, NFE2L1, NF1, USF1, RFX1, BACH1, CUX1, POU2F1, CREB1, NFYA, HNF1A, TCF3, ARNT, STAT3, FOXO1, PML, ACLY, HNF4A, LSS, LAMC1, APP, CDKN1A, MTA3, PTEN, E2F4, SCAP, PCM1, HDAC10, LPIN1, WT1, KRAS, SIRT1, RRP1B, MLXIPL, SLC2A1, ATM, PPP3CA, ITGAV, PNPLA2, VEGFA, TOPBP1, E2F3, IDH2, ABCA1, ALG2, IQCB1, MBNL2, EIF2B3, ACOT8, and SLC25A10). A prior regulatory network was obtained by associating the enriched transcription factors to the respective targets, and including regulations obtained in the TRANSFAC [85] and RegNetwork [86] databases, involving the novel set of 258 genes/proteins. In the end, a prior network of 3082 regulations between 258 genes/proteins was obtained (1877 regulations from DAVID, 232 regulations from IPA, 938 regulations from TRANSFAC, 551 regulations from RegNetwork).

#### Network inference from expression data

A regulatory network was inferred in the RNA-seq and proteomic datasets separately. In the RNA-seq data, fold change values were used (the minimum RPKM was set to 0.1). Inference was done on 6 samples (of fold change values). On both datasets, the data was log_2_ transformed and the expression of each gene/protein was divided by its standard deviation. In both datasets, network inference was done on a variable scoring manner. For each gene/protein, that gene/protein is considered a target variable, and all other genes/proteins are scored with respect to their predictive value towards it. In the proteomics dataset, the inference was directed, making use of the fact that different time points were used. In this case, the target variable takes the form “4h#1, 4h#2, 16h#1, 16h#2, 24h#1, 24h#2”. The predictor variables take the form “0h#1, 0h#2, 4h#1, 4h#2, 16h#1, 16h#2”. In the RNA-seq dataset, the inference was undirected, and the regulation score between two genes was the maximum of the two scores obtained when each of the genes was considered as target.

A random forest algorithm was used to score predictors of a target variable. A similar approach has been proposed in GENIE3 [87]. This was implemented in R using the package “randomForest” RF [88]. The number of trees was set to 20,000 and the number of variables randomly sampled as candidates at each split was set to 244/3. The adopted score (variable importance) is the total decrease in node impurities from splitting on the variable, averaged over all trees (node impurity measured by the residual sum of squares). A null distribution of random scores was obtained by shuffling the data and repeating the network inference procedure. Using this distribution, original regulation scores were associated to a *p*-value. Regulations (edges) were selected if *p* < 0.001 or alternatively if *p* < 0.05 and the regulation was present in the prior network. This analysis was performed for the 2 datasets (RNA-seq and proteomics) separately. The two obtained networks were then merged and a final network of 416 regulations involving 190 genes/proteins was obtained.

### Treatments

For validation and functional studies, INS-1E cells and dispersed human islets were exposed in independent experiments to 0.5 mM palmitate precomplexed to 0.67% FFA-free BSA for 24 h. For these experiments, human islets were cultured in the same medium as described above (see section human islets and rodent β-cells). INS-1E cells used for functional studies were authenticated by DNA bar-coding of COX subunit 1 on August 2017 and periodically tested for Mycoplasma infection. They were cultured in RPMI 1640 medium complemented as described above but containing 5% FBS, which was lowered to 1% during palmitate exposure. Exposure to palmitate (0.5 mM) in the presence of 1% charcoal-absorbed BSA or precomplexed to 0.67% FFA-free BSA results in similar unbound FFA concentrations [81]. BCH (2-Amino-2-norbornanecarboxylic acid) was used to inhibit the system L of amino-acid transporters at a concentration of 10 mM. The ER stressor CPA was used at 25 μM for 16 h. All compounds were from Sigma-Aldrich.

### RNA interference

The siRNAs are listed in Supplementary Table 3. Allstar Negative Control siRNA (siCT, Qiagen) was used as negative control. Transfection was performed using 30 nM siRNA and Lipofectamine RNAiMAX (Invitrogen-Life Technologies) as described [89]. Cells were transfected for 48 h.

### Assessment of β-cell apoptosis

Apoptotic cells were identified and counted by fluorescence microscopy after propidium iodide (5 μg/ml) and Hoechst 33342 (10 μg/ml) staining (Sigma-Aldrich) [90]. At least 400 cells were counted per experimental condition by two investigators, one of them unaware of the conditions, with an agreement between them of > 90%.

### qPCR

Poly(A)^+^ RNA was isolated using the dynabeads mRNA DIRECT kit (Invitrogen) and reverse transcribed. qPCR was performed on a Rotor-Gene Q (Qiagen) or a MyiQ2 (Bio-Rad) instrument and the amplicons were quantified as copies/μl using a standard curve. Expression was corrected for the reference genes glyceraldehyde-3-phosphate dehydrogenase (Gapdh) for rat and β-actin (ACTB) for human cells. Primer sequences are listed in Supplementary Table 4.

### Glucose-stimulated insulin secretion

INS-1E cells were pre-incubated for 1 h in RPMI GlutaMAX-I medium (0 mM glucose, Life Technologies) and for 30 min in Krebs-Ringer solution. Cells were then sequentially exposed to Krebs-Ringer containing 1.67 mM glucose, 16.7 mM glucose or 30 mM KCl for 1 h. Insulin was measured using the rat insulin ELISA (Mercodia, Uppsala, Sweden) in cell-free supernatant and acid ethanol-extracted cell lysates. Results were normalized to total protein content (assayed by Bradford).

### Statistical analysis in functional experiments

Data are shown as Tukey boxplots. Comparisons between gene expression data in treated and untreated conditions were performed by ratio t-test. Multiple comparisons between groups were performed by ANOVA followed by Sidak’s or Dunnett’s post hoc test. For gene expression data, the same tests were applied after logarithmic transformation of the data. A *p*-value< 0.05 was considered statistically significant.

## Supplementary information

**Additional file 1 Supplementary Fig. 1**. Theoretical RRHO maps. To exemplify and facilitate interpretation of the RRHO plots, RRHO maps were generated for 3 different hypothetical conditions: (A) Identical gene expression changes in two unrelated transcriptome datasets X and Y that generate perfect overlap. The red color along the diagonal indicates highly significant overlap; the blue color shows that no overlap exists between upregulated genes in X and downregulated genes in Y (upper left corner), and vice versa; (B) Identical gene expression changes among the 10% most up- or downregulated genes in the two datasets that result in highly significant overlap in the bottom left (genes similarly upregulated in both X and Y) and upper right corner (genes similarly downregulated in X and Y); (C) Two random datasets that generate no overlap (indicated by the blue color).

**Additional file 2 Supplementary Fig. 2**. CREB3L2 deficiency impairs glucose-stimulated insulin secretion. CREB3L2 mRNA expression measured by qRT-PCR in INS-1E cells (A) and human islets (B) exposed to palmitate for 24 h. (C-D) Human islet cells were transfected with CREB3L2 siRNA or control siRNA (siCT) and treated with palmitate for 24 h. (C) Apoptosis evaluated by DNA-binding dyes. (D) CREB3L2 mRNA expression measured by qPCR. (E-G) INS-1E cells were transfected with control siRNA or two Creb3l2 siRNAs. (E) Creb3l2 mRNA expression measured by qPCR. (F) Insulin secretion after incubation with 1.7 mM and 16.7 mM glucose and (G) insulin content following Creb3l2 knockdown. Insulin secretion and content were measured by ELISA and corrected by total protein content. Data are from 4 to 7 independent experiments. *p < 0.05, **p < 0.01 vs siCT transfected cells or as indicated. #*p <* 0.05, ##*p <* 0.01 for palmitate-treated vs control-treated cells.

**Additional file 3 Supplementary Fig. 3**. The amino-acid transporters SLC7A1 and SLC7A5 are upregulated during ER stress but do not mediate ER stress-induced cell death. INS-1E cells were transfected with Slc7a1 siRNA (A-D) or control siRNA (siCT) and then exposed to palmitate for 24 h (A-B) or the ER stressor CPA for 16 h (C-D). (E-G) Slc7a5 activity was inhibited in INS-1E cells by Slc7a5 siRNA (E-F) or by the chemical L-amino-acid transport inhibitor BCH (G), and cells were exposed to palmitate for 24 h. Slc7a1 (A) and Slc7a5 (E) mRNA expression assayed by qPCR. (C, D, G) Apoptosis evaluated by DNA-binding dyes. Data are from 4 to 5 independent experiments. *p < 0.05, **p < 0.01 and ***p < 0.001 vs siCT transfected cells. #*p <* 0.05, ##*p <* 0.01 and *###p <* 0.001 vs non-treated cells.

**Additional file 4 Supplementary Fig. 4.** BACH1 knockdown stimulates insulin secretion. BACH1 mRNA expression assayed by qPCR in INS-1E cells (A) and human islets (B) exposed to palmitate for 24 h. (C-D) Human islet cells were transfected with BACH1 siRNA or control siRNA (siCT) and treated with palmitate for 24 h. (C) BACH1 mRNA expression measured by qPCR. (D) Apoptosis evaluated by DNA-binding dyes. (E-I) INS-1E cells were transfected with control siRNA or two different Bach1 siRNAs. mRNA expression measured by qPCR of Bach1 (E) and heme oxygenase 1 (Hmox1) (F), a transcriptional target of Bach1. Insulin content (G) and insulin secretion after incubation with 1.7 mM and 16.7 mM glucose (H) or 1.7 mM glucose and 1.7 mM glucose plus 30 mM KCl (I). Insulin secretion and content were measured by ELISA and corrected by total protein content. Data are from 4 to 7 independent experiments. *p < 0.05, **p < 0.01, ***p < 0.001 and ****p < 0.0001 vs siCT transfected cells. #*p <* 0.05, ##*p <* 0.01 for palmitate-treated vs control-treated cells.

**Additional file 5 Supplementary Table 1**. Characteristics of the organ donors and human islet preparations used for RNA-seq. **Supplementary Table 2**. Functional classification of genes and corresponding proteins modified by palmitate. Within each functional category, genes were classified in order of fold change. The level of transcript expression in control samples is indicated in RPKM units. **Supplementary Table 3**. siRNAs. **Supplementary Table 4**. Primer sequences

## Data Availability

The transcriptome datasets generated and/or analyzed during the current study are available in NCBI’s Gene Expression Omnibus [91], series accession number GSE156109. The mass spectrometry proteomics datasets are available in the ProteomeXchange Consortium via JPOST partner repository with dataset identifier PXD020851.

## Abbreviations

CPA: Cyclopiazonic acid
DAVID: Database for Annotation, Visualization and Integrated Discovery
ER: Endoplasmic reticulum
FFA: Free fatty acids
IPA: Ingenuity Pathway Analysis
RNA-seq: RNA-sequencing
ROS: Reactive oxygen species

## References

[1] Tuomi T, Santoro N, Caprio S, Cai M, Weng J, Groop L (2014). The many faces of diabetes: a disease with increasing heterogeneity. Lancet.

[2] Abdullah A, Peeters A, de Courten M, Stoelwinder J (2010). The magnitude of association between overweight and obesity and the risk of diabetes: a meta-analysis of prospective cohort studies. Diabetes Res Clin Pract.

[3] Boden G (2008). Obesity and free fatty acids. Endocrinol Metab Clin N Am.

[4] Eizirik DL, Pasquali L, Cnop M (2020). Pancreatic β-cells in type 1 and type 2 diabetes mellitus: different pathways to failure. Nat Rev Endocrinol.

[5] Lytrivi M, Castell AL, Poitout V, Cnop M (2020). Recent insights into mechanisms of β-cell lipo- and glucolipotoxicity in type 2 diabetes. J Mol Biol.

[6] Prentki M, Peyot ML, Masiello P, Madiraju SRM (2020). Nutrient-induced metabolic stress, adaptation, detoxification, and toxicity in the pancreatic β-cell. Diabetes.

[7] Carpentier A, Mittelman SD, Bergman RN, Giacca A, Lewis GF (2000). Prolonged elevation of plasma free fatty acids impairs pancreatic beta-cell function in obese nondiabetic humans but not in individuals with type 2 diabetes. Diabetes.

[8] Kashyap S, Belfort R, Gastaldelli A, Pratipanawatr T, Berria R, Pratipanawatr W, Bajaj M, Mandarino L, DeFronzo R, Cusi K (2003). A sustained increase in plasma free fatty acids impairs insulin secretion in nondiabetic subjects genetically predisposed to develop type 2 diabetes. Diabetes.

[9] Kaiyala KJ, Prigeon RL, Kahn SE, Woods SC, Porte D, Schwartz MW (1999). Reduced beta-cell function contributes to impaired glucose tolerance in dogs made obese by high-fat feeding. Am J Phys.

[10] Gargani S, Thevenet J, Yuan JE, Lefebvre B, Delalleau N, Gmyr V, Hubert T, Duhamel A, Pattou F, Kerr-Conte J (2013). Adaptive changes of human islets to an obesogenic environment in the mouse. Diabetologia.

[11] Shimabukuro M, Zhou YT, Levi M, Unger RH (1998). Fatty acid-induced beta cell apoptosis: a link between obesity and diabetes. Proc Natl Acad Sci U S A.

[12] Ma W, Wu JH, Wang Q, Lemaitre RN, Mukamal KJ, Djousse L, King IB, Song X, Biggs ML, Delaney JA (2015). Prospective association of fatty acids in the de novo lipogenesis pathway with risk of type 2 diabetes: the cardiovascular health study. Am J Clin Nutr.

[13] Wang L, Folsom AR, Zheng ZJ, Pankow JS, Eckfeldt JH, Investigators AS (2003). Plasma fatty acid composition and incidence of diabetes in middle-aged adults: the atherosclerosis risk in communities (ARIC) study. Am J Clin Nutr.

[14] Forouhi NG, Koulman A, Sharp SJ, Imamura F, Kröger J, Schulze MB, Crowe FL, Huerta JM, Guevara M, Beulens JWJ (2014). Differences in the prospective association between individual plasma phospholipid saturated fatty acids and incident type 2 diabetes: the EPIC-InterAct case-cohort study. Lancet Diabetes Endocrinol.

[15] Cunha DA, Hekerman P, Ladrière L, Bazarra-Castro A, Ortis F, Wakeham MC, Moore F, Rasschaert J, Cardozo AK, Bellomo E (2008). Initiation and execution of lipotoxic ER stress in pancreatic beta-cells. J Cell Sci.

[16] Zhou YP, Grill VE (1994). Long-term exposure of rat pancreatic islets to fatty acids inhibits glucose-induced insulin secretion and biosynthesis through a glucose fatty acid cycle. J Clin Invest.

[17] Roomp K, Kristinsson H, Schvartz D, Ubhayasekera K, Sargsyan E, Manukyan L, Chowdhury A, Manell H, Satagopam V, Groebe K (2017). Combined lipidomic and proteomic analysis of isolated human islets exposed to palmitate reveals time-dependent changes in insulin secretion and lipid metabolism. PLoS One.

[18] Sargsyan E, Cen J, Roomp K, Schneider R, Bergsten P (2018). Identification of early biological changes in palmitate-treated isolated human islets. BMC Genomics.

[19] Weir GC (2020). Glucolipotoxicity, beta-cells, and diabetes: the emperor has no clothes. Diabetes.

[20] Wang Z, Gerstein M, Snyder M (2009). RNA-Seq: a revolutionary tool for transcriptomics. Nat Rev Genet.

[21] Wang X, Liu Q, Zhang B (2014). Leveraging the complementary nature of RNA-Seq and shotgun proteomics data. Proteomics.

[22] Cnop M, Abdulkarim B, Bottu G, Cunha DA, Igoillo-Esteve M, Masini M, Turatsinze J-V, Griebel T, Villate O, Santin I (2014). RNA sequencing identifies dysregulation of the human pancreatic islet transcriptome by the saturated fatty acid palmitate. Diabetes.

[23] Cunha DA, Igoillo-Esteve M, Gurzov EN, Germano CM, Naamane N, Marhfour I, Fukaya M, Vanderwinden JM, Gysemans C, Mathieu C (2012). Death protein 5 and p53-upregulated modulator of apoptosis mediate the endoplasmic reticulum stress-mitochondrial dialog triggering lipotoxic rodent and human beta-cell apoptosis. Diabetes.

[24] Plaisier SB, Taschereau R, Wong JA, Graeber TG (2010). Rank-rank hypergeometric overlap: identification of statistically significant overlap between gene-expression signatures. Nucleic Acids Res.

[25] Sturek JM, Castle JD, Trace AP, Page LC, Castle AM, Evans-Molina C, Parks JS, Mirmira RG, Hedrick CC (2010). An intracellular role for ABCG1-mediated cholesterol transport in the regulated secretory pathway of mouse pancreatic beta cells. J Clin Invest.

[26] Laybutt DR, Preston AM, Akerfeldt MC, Kench JG, Busch AK, Biankin AV, Biden TJ (2007). Endoplasmic reticulum stress contributes to beta cell apoptosis in type 2 diabetes. Diabetologia.

[27] Asada R, Kanemoto S, Kondo S, Saito A, Imaizumi K (2011). The signalling from endoplasmic reticulum-resident bZIP transcription factors involved in diverse cellular physiology. J Biochem.

[28] Izumi S, Saito A, Kanemoto S, Kawasaki N, Asada R, Iwamoto H, Oki M, Miyagi H, Ochi M, Imaizumi K (2012). The endoplasmic reticulum stress transducer BBF2H7 suppresses apoptosis by activating the ATF5-MCL1 pathway in growth plate cartilage. J Biol Chem.

[29] Kondo S, Saito A, Hino S-I, Murakami T, Ogata M, Kanemoto S, Nara S, Yamashita A, Yoshinaga K, Hara H (2007). BBF2H7, a novel transmembrane bZIP transcription factor, is a new type of endoplasmic reticulum stress transducer. Mol Cell Biol.

[30] Zhang B, Cunningham MA, Nichols WC, Bernat JA, Seligsohn U, Pipe SW, McVey JH, Schulte-Overberg U, de Bosch NB, Ruiz-Saez A (2003). Bleeding due to disruption of a cargo-specific ER-to-Golgi transport complex. Nat Genet.

[31] Preston AM, Gurisik E, Bartley C, Laybutt DR, Biden TJ (2009). Reduced endoplasmic reticulum (ER)-to-Golgi protein trafficking contributes to ER stress in lipotoxic mouse beta cells by promoting protein overload. Diabetologia.

[32] Hansen HG, Schmidt JD, Soltoft CL, Ramming T, Geertz-Hansen HM, Christensen B, Sorensen ES, Juncker AS, Appenzeller-Herzog C, Ellgaard L (2012). Hyperactivity of the Ero1alpha oxidase elicits endoplasmic reticulum stress but no broad antioxidant response. J Biol Chem.

[33] Wortham NC, Proud CG (2015). eIF2B: recent structural and functional insights into a key regulator of translation. Biochem Soc Trans.

[34] Saeki K, Yasugi E, Okuma E, Breit SN, Nakamura M, Toda T, Kaburagi Y, Yuo A (2005). Proteomic analysis on insulin signaling in human hematopoietic cells: identification of CLIC1 and SRp20 as novel downstream effectors of insulin. Am J Physiol Endocrinol Metab.

[35] Krokowski D, Han J, Saikia M, Majumder M, Yuan CL, Guan B-J, Bevilacqua E, Bussolati O, Bröer S, Arvan P (2013). A self-defeating anabolic program leads to β-cell apoptosis in endoplasmic reticulum stress-induced diabetes via regulation of amino acid flux. J Biol Chem.

[36] Natalicchio A, Biondi G, Marrano N, Labarbuta R, Tortosa F, Spagnuolo R, D'Oria R, Carchia E, Leonardini A, Cignarelli A (2016). Long-term exposure of pancreatic beta-cells to Palmitate results in SREBP-1C-dependent decreases in GLP-1 receptor signaling via CREB and AKT and insulin secretory response. Endocrinology.

[37] Huang da W, Sherman BT, Lempicki RA (2009). Bioinformatics enrichment tools: paths toward the comprehensive functional analysis of large gene lists. Nucleic Acids Res.

[38] Huang da W, Sherman BT, Lempicki RA (2009). Systematic and integrative analysis of large gene lists using DAVID bioinformatics resources. Nat Protoc.

[39] Auro K, Kristiansson K, Zethelius B, Berne C, Lannfelt L, Taskinen MR, Jauhiainen M, Perola M, Peltonen L, Syvanen AC (2008). USF1 gene variants contribute to metabolic traits in men in a longitudinal 32-year follow-up study. Diabetologia.

[40] Meex SJ, van Vliet-Ostaptchouk JV, van der Kallen CJ, van Greevenbroek MM, Schalkwijk CG, Feskens EJ, Blaak EE, Wijmenga C, Hofker MH, Stehouwer CD (2008). Upstream transcription factor 1 (USF1) in risk of type 2 diabetes: association study in 2000 Dutch Caucasians. Mol Genet Metab.

[41] Ng MC, Miyake K, So WY, Poon EW, Lam VK, Li JK, Cox NJ, Bell GI, Chan JC (2005). The linkage and association of the gene encoding upstream stimulatory factor 1 with type 2 diabetes and metabolic syndrome in the Chinese population. Diabetologia.

[42] Najmi LA, Aukrust I, Flannick J, Molnes J, Burtt N, Molven A, Groop L, Altshuler D, Johansson S, Bjorkhaug L (2017). Functional investigations of HNF1A identify rare variants as risk factors for type 2 diabetes in the general population. Diabetes.

[43] Kim-Muller JY, Zhao S, Srivastava S, Mugabo Y, Noh HL, Kim YR, Madiraju SR, Ferrante AW, Skolnik EY, Prentki M (2014). Metabolic inflexibility impairs insulin secretion and results in MODY-like diabetes in triple FoxO-deficient mice. Cell Metab.

[44] Kitamura T (2013). The role of FOXO1 in beta-cell failure and type 2 diabetes mellitus. Nat Rev Endocrinol.

[45] Warnatz HJ, Schmidt D, Manke T, Piccini I, Sultan M, Borodina T, Balzereit D, Wruck W, Soldatov A, Vingron M (2011). The BTB and CNC homology 1 (BACH1) target genes are involved in the oxidative stress response and in control of the cell cycle. J Biol Chem.

[46] Hall E, Volkov P, Dayeh T, Bacos K, Ronn T, Nitert MD, Ling C (2014). Effects of palmitate on genome-wide mRNA expression and DNA methylation patterns in human pancreatic islets. BMC Med.

[47] Busch AK, Gurisik E, Cordery DV, Sudlow M, Denyer GS, Laybutt DR, Hughes WE, Biden TJ (2005). Increased fatty acid desaturation and enhanced expression of stearoyl coenzyme a desaturase protects pancreatic beta-cells from lipoapoptosis. Diabetes.

[48] Malmgren S, Spegel P, Danielsson AP, Nagorny CL, Andersson LE, Nitert MD, Ridderstrale M, Mulder H, Ling C (2013). Coordinate changes in histone modifications, mRNA levels, and metabolite profiles in clonal INS-1 832/13 beta-cells accompany functional adaptations to lipotoxicity. J Biol Chem.

[49] Battle A, Khan Z, Wang SH, Mitrano A, Ford MJ, Pritchard JK, Gilad Y (2015). Genomic variation. Impact of regulatory variation from RNA to protein. Science.

[50] Hasin Y, Seldin M, Lusis A (2017). Multi-omics approaches to disease. Genome Biol.

[51] Haider S, Pal R (2013). Integrated analysis of transcriptomic and proteomic data. Curr Genomics.

[52] Lovis P, Roggli E, Laybutt DR, Gattesco S, Yang JY, Widmann C, Abderrahmani A, Regazzi R (2008). Alterations in microRNA expression contribute to fatty acid-induced pancreatic beta-cell dysfunction. Diabetes.

[53] Ciregia F, Bugliani M, Ronci M, Giusti L, Boldrini C, Mazzoni MR, Mossuto S, Grano F, Cnop M, Marselli L (2017). Palmitate-induced lipotoxicity alters acetylation of multiple proteins in clonal β cells and human pancreatic islets. Sci Rep.

[54] Maris M, Robert S, Waelkens E, Derua R, Hernangomez MH, D'Hertog W, Cnop M, Mathieu C, Overbergh L (2013). Role of the saturated nonesterified fatty acid palmitate in beta cell dysfunction. J Proteome Res.

[55] Groebe K, Cen J, Schvartz D, Sargsyan E, Chowdhury A, Roomp K, Schneider R, Alderborn A, Sanchez JC, Bergsten P (2018). Palmitate-induced insulin Hypersecretion and later secretory decline associated with changes in protein expression patterns in human pancreatic islets. J Proteome Res.

[56] Sargsyan E, Artemenko K, Manukyan L, Bergquist J, Bergsten P (2016). Oleate protects beta-cells from the toxic effect of palmitate by activating pro-survival pathways of the ER stress response. Biochim Biophys Acta.

[57] Rubi B, Antinozzi PA, Herrero L, Ishihara H, Asins G, Serra D, Wollheim CB, Maechler P, Hegardt FG (2002). Adenovirus-mediated overexpression of liver carnitine palmitoyltransferase I in INS1E cells: effects on cell metabolism and insulin secretion. Biochem J.

[58] Cantley J, Davenport A, Vetterli L, Nemes NJ, Whitworth PT, Boslem E, Thai LM, Mellett N, Meikle PJ, Hoehn KL (2019). Disruption of beta cell acetyl-CoA carboxylase-1 in mice impairs insulin secretion and beta cell mass. Diabetologia.

[59] Choi SE, Jung IR, Lee YJ, Lee SJ, Lee JH, Kim Y, Jun HS, Lee KW, Park CB, Kang Y (2011). Stimulation of lipogenesis as well as fatty acid oxidation protects against palmitate-induced INS-1 beta-cell death. Endocrinology.

[60] Wente W, Brenner MB, Zitzer H, Gromada J, Efanov AM (2007). Activation of liver X receptors and retinoid X receptors induces growth arrest and apoptosis in insulin-secreting cells. Endocrinology.

[61] Martinez SC, Tanabe K, Cras-Meneur C, Abumrad NA, Bernal-Mizrachi E, Permutt MA (2008). Inhibition of Foxo1 protects pancreatic islet beta-cells against fatty acid and endoplasmic reticulum stress-induced apoptosis. Diabetes.

[62] Talchai C, Xuan S, Lin HV, Sussel L, Accili D (2012). Pancreatic beta cell dedifferentiation as a mechanism of diabetic beta cell failure. Cell.

[63] Tian G, Sol ER, Xu Y, Shuai H, Tengholm A (2015). Impaired cAMP generation contributes to defective glucose-stimulated insulin secretion after long-term exposure to palmitate. Diabetes.

[64] Kwon G, Pappan KL, Marshall CA, Schaffer JE, McDaniel ML (2004). cAMP dose-dependently prevents palmitate-induced apoptosis by both protein kinase A- and cAMP-guanine nucleotide exchange factor-dependent pathways in beta-cells. J Biol Chem.

[65] Feliciello A, Gottesman ME, Avvedimento EV (2001). The biological functions of A-kinase anchor proteins. J Mol Biol.

[66] Anton KA, Sinclair J, Ohoka A, Kajita M, Ishikawa S, Benz PM, Renne T, Balda M, Jorgensen C, Matter K (2014). PKA-regulated VASP phosphorylation promotes extrusion of transformed cells from the epithelium. J Cell Sci.

[67] Natalicchio A, Labarbuta R, Tortosa F, Biondi G, Marrano N, Peschechera A, Carchia E, Orlando MR, Leonardini A, Cignarelli A (2013). Exendin-4 protects pancreatic beta cells from palmitate-induced apoptosis by interfering with GPR40 and the MKK4/7 stress kinase signalling pathway. Diabetologia.

[68] Campbell JE, Drucker DJ (2013). Pharmacology, physiology, and mechanisms of incretin hormone action. Cell Metab.

[69] Graciano MF, Valle MM, Kowluru A, Curi R, Carpinelli AR (2011). Regulation of insulin secretion and reactive oxygen species production by free fatty acids in pancreatic islets. Islets.

[70] Davudian S, Mansoori B, Shajari N, Mohammadi A, Baradaran B (2016). BACH1, the master regulator gene: a novel candidate target for cancer therapy. Gene.

[71] Kondo K, Ishigaki Y, Gao J, Yamada T, Imai J, Sawada S, Muto A, Oka Y, Igarashi K, Katagiri H (2013). Bach1 deficiency protects pancreatic beta-cells from oxidative stress injury. Am J Physiol Endocrinol Metab.

[72] Guasch-Ferre M, Hruby A, Toledo E, Clish CB, Martinez-Gonzalez MA, Salas-Salvado J, Hu FB (2016). Metabolomics in Prediabetes and diabetes: a systematic review and meta-analysis. Diabetes Care.

[73] Pascoe J, Hollern D, Stamateris R, Abbasi M, Romano LC, Zou B, O'Donnell CP, Garcia-Ocana A, Alonso LC (2012). Free fatty acids block glucose-induced beta-cell proliferation in mice by inducing cell cycle inhibitors p16 and p18. Diabetes.

[74] Kulkarni RN, Mizrachi EB, Ocana AG, Stewart AF (2012). Human beta-cell proliferation and intracellular signaling: driving in the dark without a road map. Diabetes.

[75] Cnop M, Hughes SJ, Igoillo-Esteve M, Hoppa MB, Sayyed F, van de Laar L, Gunter JH, de Koning EJ, Walls GV, Gray DW (2010). The long lifespan and low turnover of human islet beta cells estimated by mathematical modelling of lipofuscin accumulation. Diabetologia.

[76] Perl S, Kushner JA, Buchholz BA, Meeker AK, Stein GM, Hsieh M, Kirby M, Pechhold S, Liu EH, Harlan DM (2010). Significant human beta-cell turnover is limited to the first three decades of life as determined by in vivo thymidine analog incorporation and radiocarbon dating. J Clin Endocrinol Metab.

[77] Eizirik DL, Pipeleers DG, Ling Z, Welsh N, Hellerstrom C, Andersson A (1994). Major species differences between humans and rodents in the susceptibility to pancreatic beta-cell injury. Proc Natl Acad Sci U S A.

[78] Martens GA (2015). Species-related differences in the proteome of rat and human pancreatic Beta cells. J Diabetes Res.

[79] Nica AC, Ongen H, Irminger JC, Bosco D, Berney T, Antonarakis SE, Halban PA, Dermitzakis ET (2013). Cell-type, allelic, and genetic signatures in the human pancreatic beta cell transcriptome. Genome Res.

[80] Weir GC, Marselli L, Marchetti P, Katsuta H, Jung MH, Bonner-Weir S (2009). Towards better understanding of the contributions of overwork and glucotoxicity to the beta-cell inadequacy of type 2 diabetes. Diabetes Obes Metab.

[81] Oliveira AF, Cunha DA, Ladriere L, Igoillo-Esteve M, Bugliani M, Marchetti P, Cnop M (2015). In vitro use of free fatty acids bound to albumin: a comparison of protocols. Biotechniques.

[82] Merglen A, Theander S, Rubi B, Chaffard G, Wollheim CB, Maechler P (2004). Glucose sensitivity and metabolism-secretion coupling studied during two-year continuous culture in INS-1E insulinoma cells. Endocrinology.

[83] Ross PL, Huang YN, Marchese JN, Williamson B, Parker K, Hattan S, Khainovski N, Pillai S, Dey S, Daniels S (2004). Multiplexed protein quantitation in Saccharomyces cerevisiae using amine-reactive isobaric tagging reagents. Mol Cell Proteomics.

[84] Cahill KM, Huo Z, Tseng GC, Logan RW, Seney ML (2018). Improved identification of concordant and discordant gene expression signatures using an updated rank-rank hypergeometric overlap approach. Sci Rep.

[85] Matys V, Kel-Margoulis OV, Fricke E, Liebich I, Land S, Barre-Dirrie A, Reuter I, Chekmenev D, Krull M, Hornischer K (2006). TRANSFAC and its module TRANSCompel: transcriptional gene regulation in eukaryotes. Nucleic Acids Res.

[86] Liu ZP, Wu C, Miao H, Wu H. RegNetwork: an integrated database of transcriptional and post-transcriptional regulatory networks in human and mouse. Database (Oxford). 2015;2015. 10.1093/database/bav095 PMC4589691 26424082

[87] Huynh-Thu VA, Irrthum A, Wehenkel L, Geurts P. Inferring regulatory networks from expression data using tree-based methods. PLoS One. 2010;5(9). 10.1371/journal.pone.0012776 PMC2946910 20927193

[88] Matthew LAW (2002). Classification and regression by randomForest. R News.

[89] Moore F, Cunha DA, Mulder H, Eizirik DL (2012). Use of RNA interference to investigate cytokine signal transduction in pancreatic beta cells. Methods Mol Biol.

[90] Cnop M, Ladriere L, Hekerman P, Ortis F, Cardozo AK, Dogusan Z, Flamez D, Boyce M, Yuan J, Eizirik DL (2007). Selective inhibition of eukaryotic translation initiation factor 2 alpha dephosphorylation potentiates fatty acid-induced endoplasmic reticulum stress and causes pancreatic beta-cell dysfunction and apoptosis. J Biol Chem.

[91] Edgar R, Domrachev M, Lash AE (2002). Gene expression omnibus: NCBI gene expression and hybridization array data repository. Nucleic Acids Res.

