# Supplementary figures and images for: Combined transcriptome and proteome profiling of the pancreatic β-cell response to palmitate unveils key pathways of β-cell lipotoxicity
Source: BMC Genomics. 2020 Aug 26;21:590. doi: 10.1186/s12864-020-07003-0 (PMC7448506; doi:10.1186/s12864-020-07003-0)

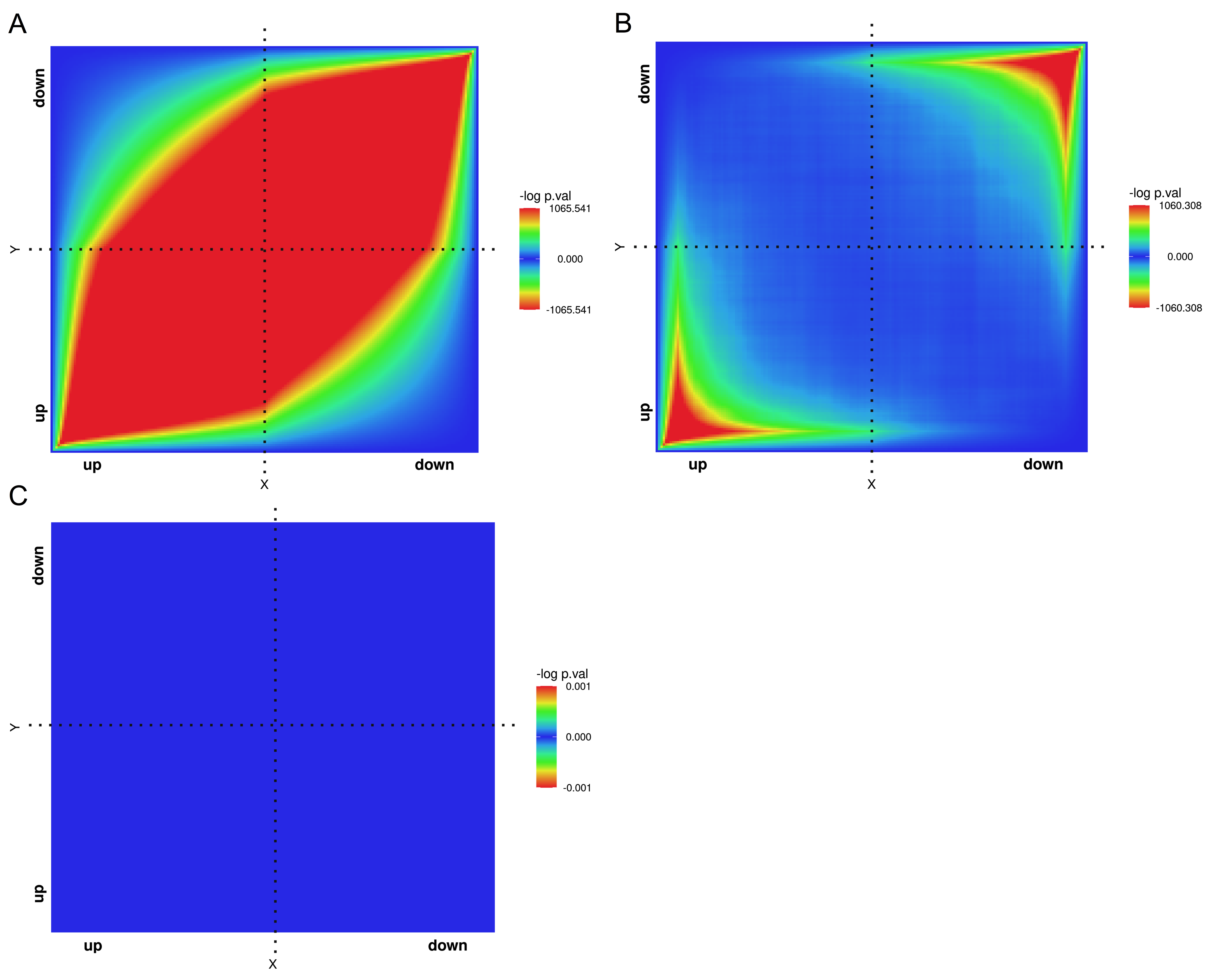

Supplement: Supplementary file 1 — Additional file 1 Supplementary Fig. 1. Theoretical RRHO maps. To exemplify and facilitate interpretation of the RRHO plots, RRHO maps were generated for 3 different hypothetical conditions: (A) Identical gene expression changes in two unrelated transcriptome datasets X and Y that generate perfect overlap. The red color along the diagonal indicates highly significant overlap; the blue color shows that no overlap exists between upregulated genes in X and downregulated genes in Y (upper left corner), and vice versa; (B) Identical gene expression changes among the 10% most up- or downregulated genes in the two datasets that result in highly significant overlap in the bottom left (genes similarly upregulated in both X and Y) and upper right corner (genes similarly downregulated in X and Y); (C) Two random datasets that generate no overlap (indicated by the blue color). [file 12864_2020_7003_MOESM1_ESM.tiff]

## Slide 1
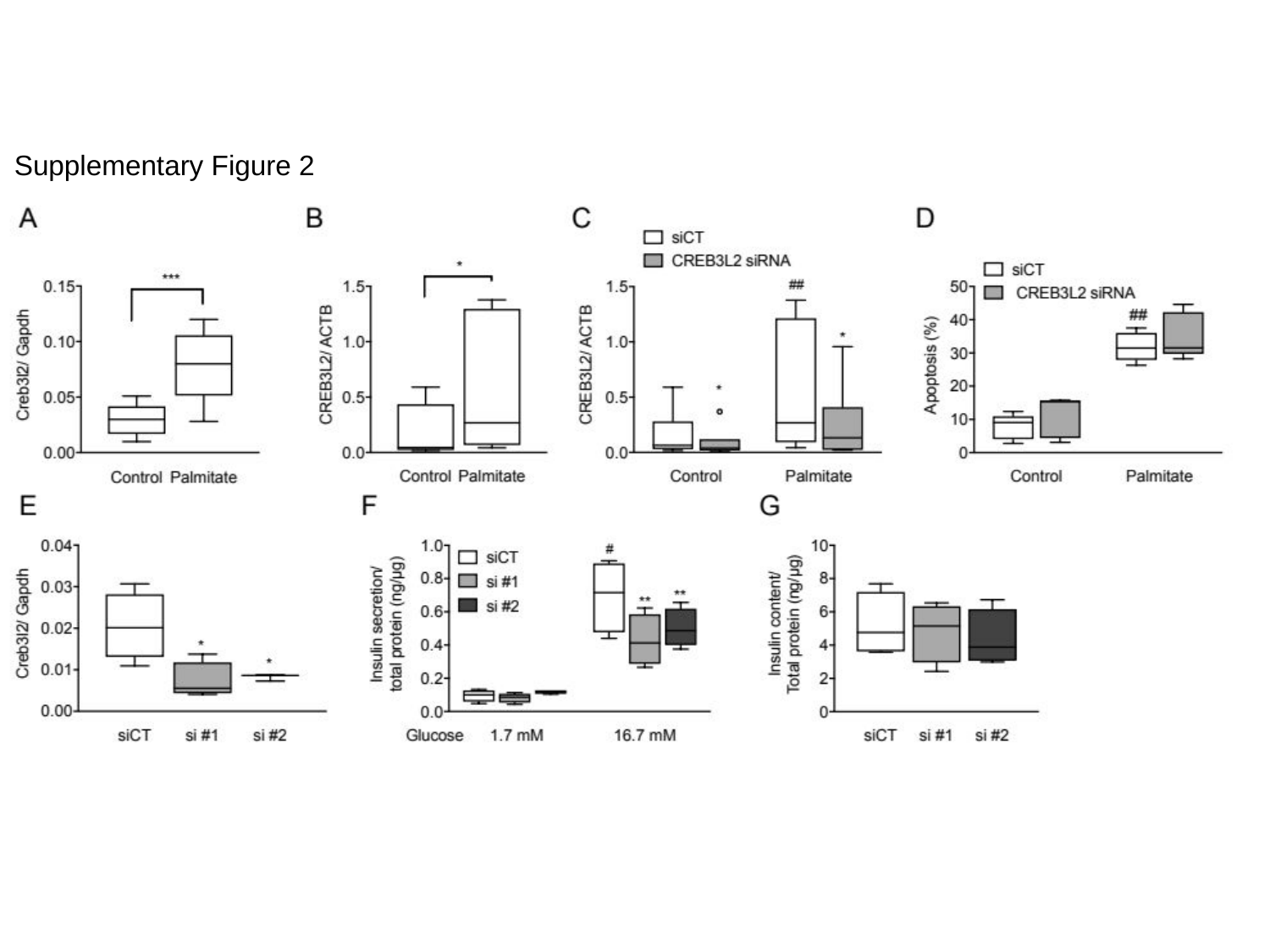

Supplementary Figure 2

Supplement: Supplementary file 2 — Additional file 2 Supplementary Fig. 2. CREB3L2 deficiency impairs glucose-stimulated insulin secretion. CREB3L2 mRNA expression measured by qRT-PCR in INS-1E cells (A) and human islets (B) exposed to palmitate for 24 h. (C-D) Human islet cells were transfected with CREB3L2 siRNA or control siRNA (siCT) and treated with palmitate for 24 h. (C) Apoptosis evaluated by DNA-binding dyes. (D) CREB3L2 mRNA expression measured by qPCR. (E-G) INS-1E cells were transfected with control siRNA or two Creb3l2 siRNAs. (E) Creb3l2 mRNA expression measured by qPCR. (F) Insulin secretion after incubation with 1.7 mM and 16.7 mM glucose and (G) insulin content following Creb3l2 knockdown. Insulin secretion and content were measured by ELISA and corrected by total protein content. Data are from 4 to 7 independent experiments. *p < 0.05, **p < 0.01 vs siCT transfected cells or as indicated. #p < 0.05, ##p < 0.01 for palmitate-treated vs control-treated cells. [file 12864_2020_7003_MOESM2_ESM.pptx]

## Slide 1
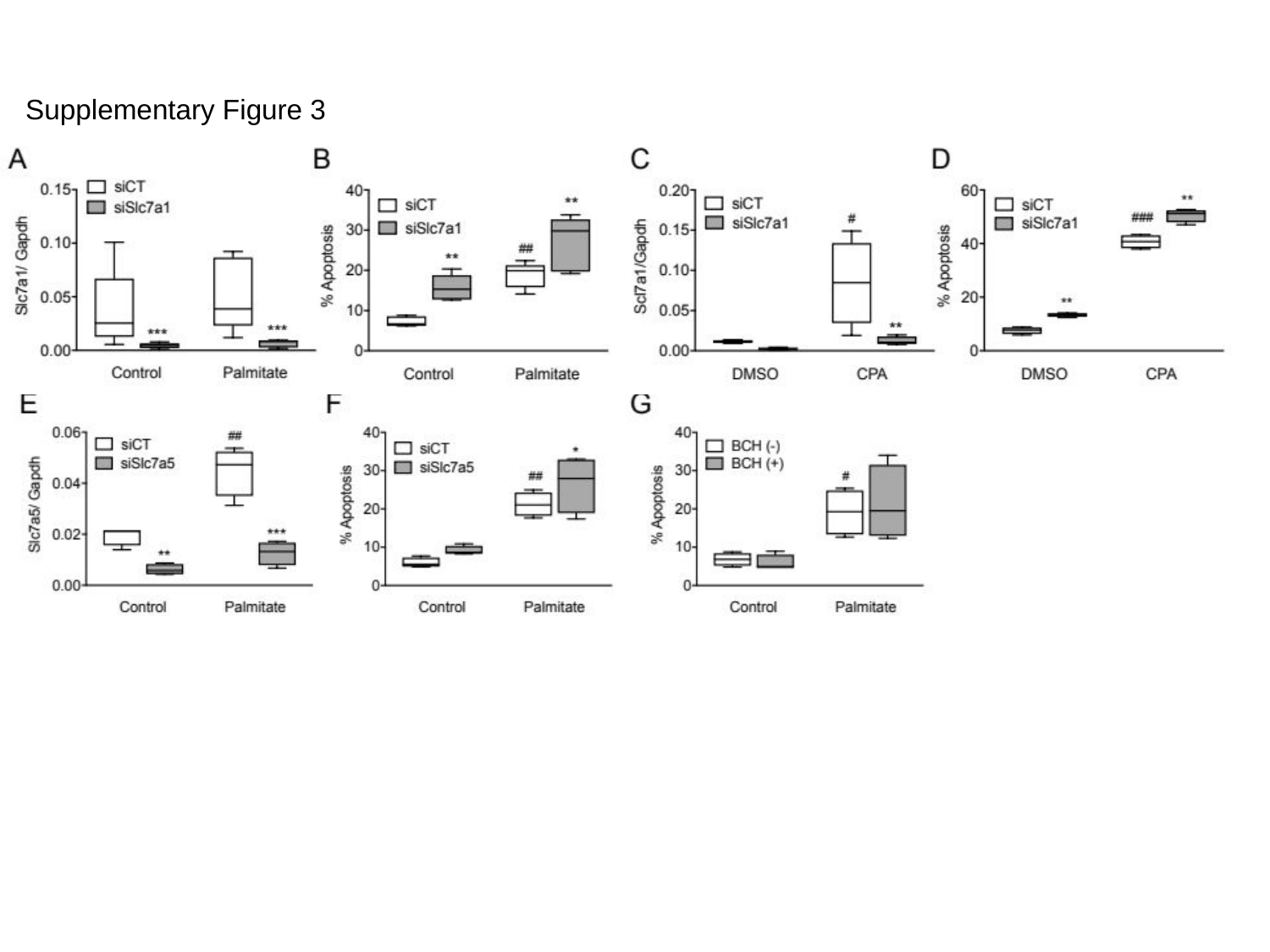

Supplementary Figure 3

Supplement: Supplementary file 3 — Additional file 3 Supplementary Fig. 3. The amino-acid transporters SLC7A1 and SLC7A5 are upregulated during ER stress but do not mediate ER stress-induced cell death. INS-1E cells were transfected with Slc7a1 siRNA (A-D) or control siRNA (siCT) and then exposed to palmitate for 24 h (A-B) or the ER stressor CPA for 16 h (C-D). (E-G) Slc7a5 activity was inhibited in INS-1E cells by Slc7a5 siRNA (E-F) or by the chemical L-amino-acid transport inhibitor BCH (G), and cells were exposed to palmitate for 24 h. Slc7a1 (A) and Slc7a5 (E) mRNA expression assayed by qPCR. (C, D, G) Apoptosis evaluated by DNA-binding dyes. Data are from 4 to 5 independent experiments. *p < 0.05, **p < 0.01 and ***p < 0.001 vs siCT transfected cells. #p < 0.05, ##p < 0.01 and ###p < 0.001 vs non-treated cells. [file 12864_2020_7003_MOESM3_ESM.pptx]

## Slide 1
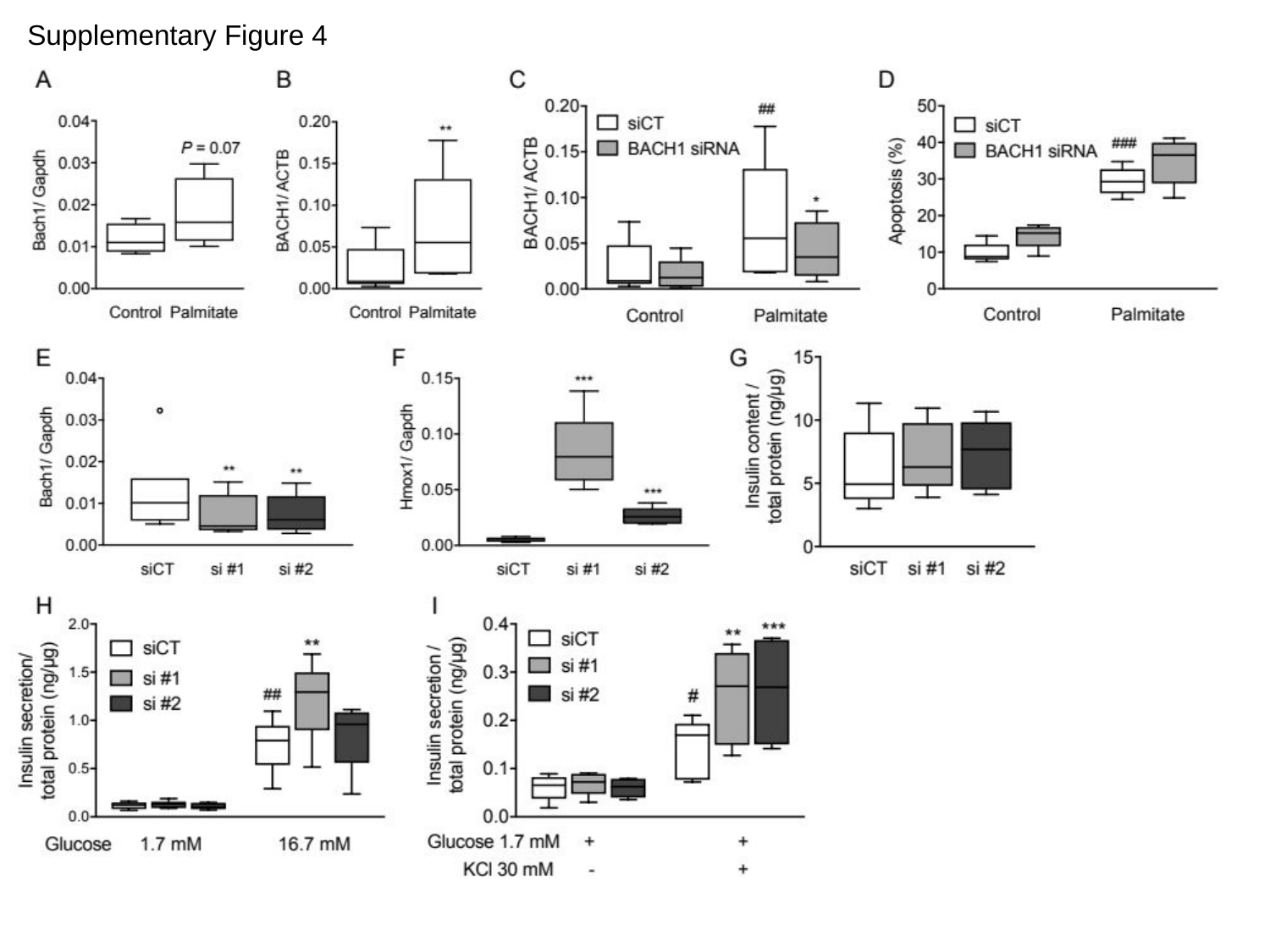

Supplementary Figure 4

Supplement: Supplementary file 4 — Additional file 4 Supplementary Fig. 4. BACH1 knockdown stimulates insulin secretion. BACH1 mRNA expression assayed by qPCR in INS-1E cells (A) and human islets (B) exposed to palmitate for 24 h. (C-D) Human islet cells were transfected with BACH1 siRNA or control siRNA (siCT) and treated with palmitate for 24 h. (C) BACH1 mRNA expression measured by qPCR. (D) Apoptosis evaluated by DNA-binding dyes. (E-I) INS-1E cells were transfected with control siRNA or two different Bach1 siRNAs. mRNA expression measured by qPCR of Bach1 (E) and heme oxygenase 1 (Hmox1) (F), a transcriptional target of Bach1. Insulin content (G) and insulin secretion after incubation with 1.7 mM and 16.7 mM glucose (H) or 1.7 mM glucose and 1.7 mM glucose plus 30 mM KCl (I). Insulin secretion and content were measured by ELISA and corrected by total protein content. Data are from 4 to 7 independent experiments. *p < 0.05, **p < 0.01, ***p < 0.001 and ****p < 0.0001 vs siCT transfected cells. #p < 0.05, ##p < 0.01 for palmitate-treated vs control-treated cells. [file 12864_2020_7003_MOESM4_ESM.pptx]
