# Supplementary material for: Combined transcriptome and proteome profiling of the pancreatic β-cell response to palmitate unveils key pathways of β-cell lipotoxicity
Source: BMC Genomics. 2020 Aug 26;21:590. doi: 10.1186/s12864-020-07003-0 (PMC7448506; doi:10.1186/s12864-020-07003-0)
Supplement: Supplementary file 5 — Additional file 5 Supplementary Table 1. Characteristics of the organ donors and human islet preparations used for RNA-seq. Supplementary Table 2. Functional classification of genes and corresponding proteins modified by palmitate. Within each functional category, genes were classified in order of fold change. The level of transcript expression in control samples is indicated in RPKM units. Supplementary Table 3. siRNAs. Supplementary Table 4. Primer sequences [file 12864_2020_7003_MOESM5_ESM.docx]

**Combined transcriptome and proteome profiling of the pancreatic β-cell response to palmitate unveils key pathways of β-cell lipotoxicity**

Maria Lytrivi^1,2^, Kassem Ghaddar^1^, Miguel Lopes^1^, Victoria Rosengren^3^, Anthony Piron^1^, Xiaoyan Yi^1^, Henrik Johansson^4^, Janne Lehtiö^4^, Mariana Igoillo-Esteve^1^, Daniel A Cunha^1^, Lorella Marselli^5^, Piero Marchetti^5^, Henrik Ortsäter^3^, Decio L Eizirik^1^, Miriam Cnop^1,2^

^1^ULB Center for Diabetes Research, Université Libre de Bruxelles, Brussels, Belgium, ^2^Division of Endocrinology, Erasmus Hospital, Université Libre de Bruxelles, Brussels, Belgium, ^3^Diabetes Research Unit, Department of Clinical Science and Education, Sodersjukhuset, Karolinska Institutet, Stockholm, Sweden ^4^Clinical Proteomics Mass Spectrometry, Department of Oncology-Pathology, Karolinska Institutet, Science for Life Laboratory, 171 21, Solna, Sweden, ^5^Department of Clinical and Experimental Medicine, University of Pisa, Pisa, Italy

**ADDITIONAL MATERIAL**

Additional methods:

Cell exposure and sample preparation for mass spectrometry

INS-1E cells for iTRAQ analysis were plated in 100 mm Petri dishes at a density of 2,400,000 cells per dish in 15 ml medium for 72h prior to treatment. Cells were exposed to 0.4 mM palmitate in the presence of 0.5% BSA for 0, 4, 16 and 24h. Two independent replicates were performed for each treatment. After exposure, cells were harvested by trypsinization and washed in PBS. Cells were lysed in buffer containing 4% SDS, 25 mM HEPES, 1 mM DTT and Protease inhibitory cocktail (Sigma-Aldrich), heated at 95°C for 5 min followed by sonication. The lysate was centrifuged for 30 min at 14,000 g. Samples were mixed with 8 M urea (Sigma-Aldrich), 1 mM DTT, 25 mM HEPES, pH 7.6, in a centrifugation filtering unit, 10 kDa cutoff (Nanosep Centrifugal Devices with Omega Membrane, 10 k), and centrifuged for 30 min at 14,000 g, followed by another addition of 8 M urea buffer and centrifugation. Proteins were alkylated by 50 mM iodoacetamide (Sigma-Aldrich) in 4 M urea, 25 mM HEPES, pH 7.6, for 10 min incubation at room temperature and centrifugation for 30 min at 14,000 g, followed by 2 more additions of 4 M urea and 25 mM HEPES, pH 7.6, and centrifugations for 30 min at 14,000 g. Trypsin (TPCK-treated, Applied Biosystems), 1:50, trypsin:protein, was added to the samples in 0.25 M urea, 25 mM HEPES and digested overnight at 37°C. The filter units were centrifuged for 30 min, 14,000 g, followed by another centrifugation with milli-Q water and the flow-through was collected. iTRAQ labeling of the peptides was done according to the manufacturer’s protocol (Applied Biosystems, Foster City, CA) and cleaned by strata-X-C-cartridge (Phenomenex Inc, Torrance, CA).

IPG-IEF of peptides

The iTRAQ-labeled peptides, 800 µg, were separated by immobilized pH gradient - isoelectric focusing (IPG-IEF) on a narrow range pH 3.7-4.9 strip [1]. Peptides were extracted from the strips by a prototype liquid-handling robot (GE Healthcare Bio-Sciences AB). A plastic device with 72 wells was put onto each strip and 50 µl of milli-Q water was added to each well. After 30 min incubation, the liquid was transferred to a 96-well plate and the extraction was repeated 2 more times. The extracted peptides were dried in speed vac and dissolved in 3% acetonitrile (ACN), 0.1% formic acid.

LC-ESI-LTQ-Orbitrap analysis

Before analysis on the LTQ Orbitrap Velos (Thermo Fischer Scientific, San Jose, CA), peptides were separated using an Agilent 1200 nano-LC system. Samples were trapped on a Zorbax 300SB-C18, and separated on a NTCC-360/100-5-153 (Nikkyo Technos Ltd) column using a gradient of A (3% ACN, 0.1% FA) and B (95% ACN, 0.1% FA), ranging from 3% to 40% B in 50 or 90 min with a flow of 0.4 µl/min. The LTQ Orbitrap Velos was operated in a data-dependent manner, selecting 5 precursors for sequential fragmentation by CID and HCD, and analyzed by the linear iontrap and orbitrap, respectively. The survey scan was performed in the Orbitrap at 30,000 resolution (profile mode) from 300-2000 *m/z,* using lock mass at *m/z* 445.120025, with a max injection time of 500 ms and AGC set to 1 x 10^6^ ions. For generation of HCD fragmentation spectra, a max ion injection time of 500 ms and AGC of 5 x 10^4^ were used before fragmentation at 50% normalized collision energy. For FTMS MS2 spectra, normal mass range was used, centroiding the data at 7500 resolution. Peptides for CID were accumulated for a max ion injection time of 200 ms and AGC of 3 x 10^4^, fragmented with 35% collision energy, wideband activation on, activation q 0.25, activation time 10 ms before analysis at normal scan rate and mass range in the linear iontrap. Precursors were isolated with a width of 2 *m/z* and put on the exclusion list for 90 s. Single and unassigned charge states were rejected from precursor selection.

Peptide and protein identification

All Orbitrap data were searched by Sequest-percolator under the software platform Proteome Discoverer 1.3 (Thermo Fischer Scientific) against the Uniprot rat canonical sequence protein database (27,316 entries, December 2013) using a 1% false discovery rate cutoff. A precursor mass tolerance of 10 ppm, and product mass tolerances of 0.02 Da for HCD-FTMS and 0.8 Da for CID-ITMS were used. Further settings used were: trypsin with 1 missed cleavage; iodoacetamide on cysteine and iTRAQ8plex on lysine and *N*-terminal as fixed modifications; and oxidation of methionine as variable modification. Quantification of iTRAQ8plex reporter ions was done by Proteome Discoverer on HCD-FTMS tandem mass spectra using an integration window tolerance of 20 ppm. Only unique peptides in the data set were used for quantitation.

**References**

1. Branca RM, Orre LM, Johansson HJ, Granholm V, Huss M, Perez-Bercoff A, Forshed J, Kall L, Lehtio J: **HiRIEF LC-MS enables deep proteome coverage and unbiased proteogenomics**. *Nature methods* 2014, **11**(1):59-62.

**Supplementary table 1:** **Characteristics of the organ donors and human islet preparations used for RNA-seq**

| **Sex** | **Age (years)** | **BMI (kg/m^2^)** | **Cause of death** | **Purity (%)** |
| --- | --- | --- | --- | --- |
| F | 77 | 23.8 | Trauma | 45 |
| M | 40 | 26.2 | Trauma | 34 |
| F | 46 | 22.5 | CVD | 60 |
| M | 36 | 26.3 | CVD | 51 |
| M | 77 | 25.2 | CVD | 62 |
| M | 56 | 24.7 | CVD | 47 |
| Abbreviations: F: Female; M: Male; BMI: Body mass index; CVD: Cardiovascular disease. Purity indicates the percentage of β-cells in the human islet preparations as determined by immunostaining for insulin. | | | | |

**Supplementary Table 2: Functional classification of genes and corresponding proteins modified by palmitate.** Within each functional category, genes were classified in order of fold change. The level of transcript expression in control samples is indicated in RPKM units.

| **Gene name** | **Gene description** | **Median RPKM** | **Log_2_ fold change** | **Σ# PSMs** |  |  |
| --- | --- | --- | --- | --- | --- | --- |
| **Lipid metabolism** | | | |  |  |  |
| CPT1A | Carnitine palmitoyltransferase 1A | 29.021 | 0.768 | 27 |  |  |
| ACADVL | Acyl-CoA dehydrogenase, very long chain | 778.207 | 0.695 | 150 |  |  |
| LDLR | Low density lipoprotein receptor | 59.211 | 0.659 | 27 |  |  |
| ECH1 | Enoyl CoA hydratase 1, peroxisomal | 111.767 | 0.598 | 38 |  |  |
| FADS1 | Fatty acid desaturase 1 | 35.938 | 0.542 | 9 |  |  |
| ACAA2 | Acetyl-Co-acyltransferase 2 | 47.147 | 0.281 | 183 |  |  |
| HADHB | Hydroxyacyl-CoA dehydrogenase/3-ketoacyl-CoA thiolase/enoyl-CoA hydratase (trifunctional protein), beta subunit | 56.756 | 0.194 | 211 |  |  |
| ACACA | Acetyl-CoA carboxylase alpha | 20.232 | -0.137 | 349 |  |  |
| DAGLA | Diacylglycerol lipase, alpha | 2.997 | -0.237 | 6 |  |  |
| LYPLA1 | Lysophospholipase I | 19.78 | -0.303 | 47 |  |  |
| SORL1 | Sortilin-related receptor, L(DLR class) A repeats containing | 38.49 | -0.36 | 26 |  |  |
| ABCG1 | ATP-binding cassette, sub-family G, member 1 | 9.261 | -0.375 | 2 |  |  |
| DHCR24 | 24-dehydrocholesterol reductase | 39.34 | -0.403 | 20 |  |  |
| GPAM | Glycerol-3-phosphate acyltransferase mitochondrial | 1.595 | -0.498 | 11 |  |  |
| COL4A3BP | Collagen, type IV, alpha 3 (Goodpasture antigen) binding protein | 10.835 | -0.553 | 16 |  |  |
| SMPD3 | Sphingomyelin phosphodiesterase 3, neutral membrane (neutral sphingomyelinase II) | 4.86 | -0.576 | 33 |  |  |
| ST3GAL5 | ST3 beta-galactoside alpha-2,3-sialyltransferase 5 | 6.535 | -0.636 | 8 |  |  |
| **Amino acid metabolism** | |  |  |  |  |  |
| AMDHD1 | Amidohydrolase domain containing 1 | 4.377 | 0.655 | 6 |  |  |
| PYCR1 | Pyrroline-5-carboxylate reductase 1 | 17.584 | 0.529 | 41 |  |  |
| **Metabolism-miscellaneous** | | |  |  |  |  |
| MOCOS | Molybdenum cofactor sulfurase | 1.407 | 0.583 | 1 |  |  |
| GC | Group-specific component | 65.545 | -0.392 | 195 |  |  |
| **Hormones/growth factors/receptors/neuropeptides and exocytosis** | | | |  |  |  |
| EPHA2 | EPH receptor A2 | 8.705 | 0.734 | 7 |  |  |
| SYT12 | Synaptotagmin 12 | 1.526 | 0.614 | 3 |  |  |
| NUCB2 | Nucleobindin 2 | 110.493 | 0.412 | 410 |  |  |
| TBRG1 | Transforming Growth Factor Beta Regulator 1 | 23.593 | 0.127 | 14 |  |  |
| GPR107 | G protein-coupled receptor 107 | 15.178 | -0.148 | 2 |  |  |
| GLP1R | Glucagon-like peptide 1 receptor | 9.563 | -0.284 | 12 |  |  |
| EPB41L1 | Erythrocyte membrane protein band 4.1-like | 41.07 | -0.335 | 5 |  |  |
| TGFB2 | Transforming growth factor, beta 2 | 11.426 | -0.497 | 6 |  |  |
| INSRR | Insulin Receptor Related Receptor | 0.065 | -1.552 | 2 |  |  |
| **Protein synthesis/translation regulation/protein folding/endoplasmic reticulum stress** | | | |  |  |  |
| LMAN1 | Lectin, mannose-binding 1 | 49.697 | 0.744 | 326 |  |  |
| CRELD2 | Cysteine-rich with EGF-like domains 2 | 26.792 | 0.55 | 17 |  |  |
| SRPRB | Signal recognition particle receptor subunit B | 43.153 | 0.433 | 22 |  |  |
| ERO1A | Endoplasmic reticulum oxidoreductase 1 alpha | 95.752 | 0.335 | 50 |  |  |
| EIF2S2 | Eukaryotic translation initiation factor 2, subunit2 | 35.938 | 0.329 | 38 |  |  |
| EEF1A2 | Eukaryotic translation elongation factor 1 alpha 2 | 120.422 | 0.293 | 515 |  |  |
| PDIA6 | Protein disulfide isomerase family A, member 6 | 128.973 | 0.273 | 522 |  |  |
| CTIF | CBP80/20-dependent translation initiation factor | 10.46 | 0.212 | 21 |  |  |
| LMAN2 | Lectin, mannose-binding 2 | 78.7 | 0.2 | 98 |  |  |
| CREB3L2 | cAMP responsive element binding protein 3-like 2 | 19.645 | 0.191 | 6 |  |  |
| RPLP1 | Ribosomal protein, large, P1 | 971.078 | 0.157 | 26 |  |  |
| EIF2B5 | Eukaryotic translation initiation factor 2B, subunit 5 | 38.602 | -0.109 | 31 |  |  |
| EIF4EBP2 | Eukaryotic translation initiation factor 4E binding protein 2 | 14.524 | -0.21 | 38 |  |  |
| DNAJB4 | DnaJ (Hsp40) homolog, subfamily B, member 4 | 12.475 | -0.27 | 15 |  |  |
| **Posttranslational modification/ubiquitination** | | | |  |  |  |
| FUT11 | Fucosyltransferase 11 | 17.286 | 0.242 | 1 |  |  |
| UBAP1 | Ubiquitin associated protein 1 | 23.059 | 0.17 | 3 |  |  |
| RBBP7 | Retinoblastoma binding protein 7 | 41.008 | -0.168 | 62 |  |  |
| PCSK5 | Proprotein convertase subtilisin/kexin type 5 | 1.946 | -0.266 | 9 |  |  |
| ST6GAL1 | ST6 beta-galactosamide alpha-2,6-sialyltranferase 1 | 21.655 | -0.306 | 1 |  |  |
| HERC3 | HECT and RLD domain containing E3 ubiquitin protein ligase 3 | 31.154 | -0.308 | 3 |  |  |
| **Proteasome/lysosome/autophagy** | | |  |  |  |  |
| SQSTM1 | Sequestosome 1 | 404.362 | 0.678 | 59 |  |  |
| PSMD11 | Proteasome 26S Subunit, Non-ATPase 11 | 40.856 | 0.162 | 141 |  |  |
| BLOC1S1 | Biogenesis of lysosomal organelles complex-1, subunit 1 | 45.827 | -0.334 | 3 |  |  |
| **Vesicle transport** | |  |  |  |  |  |
| STX4 | Syntaxin 4 | 37.506 | 0.202 | 15 |  |  |
| YKT6 | YKT6 v-SNARE homolog | 44.722 | 0.186 | 36 |  |  |
| VAPA | VAMP (vesicle-associated membrane protein)-associated protein A | 82.17 | -0.207 | 11 |  |  |
| **Mitochondrial proteins/enzymes** | | |  |  |  |  |
| TIMM17A | Translocase of inner mitochondrial membrane 17 homolog A | 23.913 | 0.394 | 6 |  |  |
| PDP1 | Pyruvate dehydrogenase phosphatase catalytic subunit 1 | 37.333 | 0.362 | 10 |  |  |
| POLRMT | RNA Polymerase Mitochondrial | 20.576 | 0.351 | 2 |  |  |
| TOMM40 | Translocase of outer mitochondrial membrane 40 homolog | 16.451 | 0.259 | 22 |  |  |
| NDUFA6 | NADH dehydrogenase (ubiquinone) 1 alpha subcomplex, 6 | 44.556 | 0.25 | 7 |  |  |
| TIMM44 | Translocase of inner mitochondrial membrane 44 | 19.92 | 0.225 | 138 |  |  |
| ADCK3 | aarF domain containing kinase 3 | 22.774 | -0.244 | 4 |  |  |
| **Channels and transporters** | | |  |  |  |  |
| CLIC1 | Chloride intracellular channel 1 | 191.019 | 0.588 | 77 |  |  |
| SLC7A1 | Solute carrier family 7 (amino acid transporter light chain, L system), member 1 | 23.999 | 0.318 | 23 |  |  |
| MAGT1 | Magnesium transporter 1 | 29.46 | 0.237 | 7 |  |  |
| ANO10 | Anoctamin 10 | 14.372 | 0.131 | 2 |  |  |
| ATP1B1 | ATPase, Na+/K+ transporting, beta 1 polypeptide | 305.734 | 0.087 | 33 |  |  |
| SLC9A3R1 | Solute carrier family 9, subfamily A (NHE3, cation proton antiporter 3), member 3 regulator 1 | 74.826 | -0.215 | 129 |  |  |
| ABCG1 | ATP-binding cassette, sub-family G, member 1 | 38.49 | -0.375 | 2 |  |  |
| SLC39A10 | Solute carrier family 39 member 10 | 6.43 | -0.852 | 11 |  |  |
| KCNH6 | Potassium channel, voltage gated eag related subfamily H, member 6 | 3.039 | -1.247 | 12 |  |  |
| **Cytoskeleton and related proteins** | | |  |  |  |  |
| VASP | Vasodilator-stimulated phosphoprotein | 48.428 | 0.388 | 58 |  |  |
| ELMO3 | Engulfment and cell motility 3 | 17.922 | 0.256 | 7 |  |  |
| MYO5B | Myosin VB | 50.428 | 0.11 | 42 |  |  |
| PFN2 | Profilin 2 | 61.071 | -0.290 | 129 |  |  |
| SDC1 | Syndecan 1 | 17.525 | -0.3 | 3 |  |  |
| PRC1 | Protein regulator of cytokinesis 1 | 1.817 | -0.377 | 34 |  |  |
| ABLIM1 | Actin binding LIM protein family, member 1 | 38.633 | -0.392 | 1 |  |  |
| STMN1 | Stathmin 1 | 26.699 | -0.517 | 233 |  |  |
| KIF22 | Kinesin family member 22 | 3.37 | -0.524 | 16 |  |  |
| KIF11 | Kinesin family member 11 | 0.149 | -0.866 | 37 |  |  |
| MNS1 | Meiosis-specific nuclear structural 1 | 0.735 | -1.043 | 4 |  |  |
| **Peptidase/protease** | |  |  |  |  |  |
| ERAP1 | Endoplasmic reticulum aminopeptidase 1 | 23.358 | 0.357 | 75 |  |  |
| AMZ2 | Archaelysin family metallopeptidase 2 | 25.894 | -0.171 | 3 |  |  |
| BACE1 | Beta-site APP-cleaving enzyme 1 | 26.767 | -0.37 | 6 |  |  |
| **Transcription factors** | |  |  |  |  |  |
| JUND | Jun-D protooncogene | 80.924 | 0.442 | 2 |  |  |
| MAFK | v-maf musculoaponeurotic fibrosarcoma oncogene homolog K (avian) | 18.336 | 0.358 | 10 |  |  |
| HIVEP2 | Human immunodeficiency virus type I enhancer binding protein 2 | 9.93 | 0.309 | 3 |  |  |
| ETS2 | v-ets avian erythroblastosis virus E26 oncogene homolog 2 | 43.807 | 0.198 | 4 |  |  |
| CREB3L2 | cAMP responsive element binding protein 3-like 2 | 19.645 | 0.191 | 6 |  |  |
| RNF10 | Ring finger protein 10 | 120.423 | 0.167 | 2 |  |  |
| **NF-kB regulation** | |  |  |  |  |  |
| SQSTM1 | sequestosome 1 | 404.362 | 0.678 | 59 |  |  |
| TLE1 | Transducin-like enhancer of split 1 (E(sp1) homolog | 30.796 | 0.205 | 6 |  |  |
| **Chemokines/cytokines/adhesion molecules/innate immunity and related proteins** | | | |  |  |  |
| VIMP | VCP-interacting membrane protein | 46.605 | 0.628 | 46 |  |  |
| PTGR2 | Prostaglandin reductase 2 | 5.775 | -0.295 | 3 |  |  |
| ITGB1BP1 | Integrin beta 1 binding protein 1 | 18.391 | -0.303 | 4 |  |  |
| JCAD | Junctional cadherin 5 associated | 8.045 | -0.351 | 2 |  |  |
| SKAP2 | src kinase associated phosphoprotein 2 | 4.157 | -0.425 | 45 |  |  |
| LGALS2 | Lectin, galactoside-binding, soluble, 2 | 36.252 | -0.509 | 29 |  |  |
| C2CD4C | C2 calcium-dependent domain containing 4C | 0.679 | -0.802 | 19 |  |  |
| FN1 | Fibronectin 1 | 64.511 | -0.979 | 447 |  |  |
| **Signal transduction** | |  |  |  |  |  |
| GEM | GTP binding protein | 18.269 | 0.752 | 2 |  |  |
| ARL1 | ADP-ribosylation factor-like 1 | 79.852 | 0.369 | 70 |  |  |
| PDE4D | Phosphodiesterase 4 | 3.391 | 0.349 | 32 |  |  |
| GIT1 | G protein-coupled receptor kinase interacting ArfGAP 1 | 22.996 | 0.128 | 85 |  |  |
| AKAP11 | A kinase (PRKA) anchor protein 11 | 9.564 | -0.187 | 6 |  |  |
| AKAP1 | A kinase (PRKA) anchor protein 1 | 16.731 | -0.193 | 9 |  |  |
| DIXDC1 | DIX domain containing 1 | 5.267 | -0.315 | 7 |  |  |
| RASA1 | RAS p21 protein activator 1 | 10.298 | -0.348 | 54 |  |  |
| DLG2 | Discs, large homolog 2 (Drosophila) | 3.758 | -0.454 | 14 |  |  |
| OPHN1 | Oligophrenin 1 | 1.464 | -0.468 | 12 |  |  |
| AKAP7 | A kinase (PRKA) anchor protein 7 | 17.469 | -0.478 | 8 |  |  |
| ARHGEF28 | Rho guanine nucleotide exchange factor (GEF) 28 | 5.315 | -0.585 | 6 |  |  |
| **Kinases/ phosphatases** | |  |  |  |  |  |
| DUSP4 | Dual specificity phosphatase 4 | 25.854 | 0.437 | 2 |  |  |
| VRK3 | Vaccinia related kinase 3 | 18.767 | -0.31 | 8 |  |  |
| COL4A3BP | Collagen, type IV, alpha 3 (Goodpasture antigen) binding protein | 10.835 | -0.553 | 16 |  |  |
| **Transcription regulation/alternative splicing** | | | |  |  |  |
| PPAN | Peter pan homolog | 16.262 | 0.369 | 10 |  |  |
| DRAP1 | DR1-associated protein 1 | 51.236 | 0.341 | 9 |  |  |
| SNRPB | Small nuclear ribonucleoprotein polypeptides B and B1 | 37.208 | 0.278 | 31 |  |  |
| ZPR1 | ZPR1 Zinc Finger | 15.613 | 0.23 | 17 |  |  |
| HCFC1 | Host cell factor C1 | 17.887 | 0.178 | 28 |  |  |
| CREBBP | CREB binding protein | 20.06 | 0.127 | 10 |  |  |
| TARBP2 | TAR (HIV-1) RNA binding protein 2 | 11.636 | -0.255 | 8 |  |  |
| SUPT4H1 | Suppressor of Ty 4 homolog 1 | 30.288 | -0.325 | 13 |  |  |
| TCEA2 | Transcription elongation factor A (SII), 2 | 26.824 | -0.336 | 10 |  |  |
| HMGN3 | High mobility group nucleosomal binding domain 3 | 35.767 | -0.391 | 19 |  |  |
| SMARCD3 | SWI/SNF related, matrix associated, actin dependent regulator of chromatin, subfamily d, member 3 | 24.073 | -0.453 | 57 |  |  |
| ADI1 | Acireductone dioxygenase 1 | 19.653 | -0.540 | 27 |  |  |
| ZNF182 | Zinc Finger Protein 182 | 1.705 | -0.562 | 1 |  |  |
| **Epigenetic regulation** | |  |  |  |  |  |
| SMARCD3 | SWI/SNF related, matrix associated, actin dependent regulator of chromatin, subfamily d, member 3 | 24.073 | -0.453 | 57 |  |  |
| ATAD2 | ATPase family, AAA domain containing 2 | 1.221 | -0.549 | 97 |  |  |
| **Oxidative stress/DNA damage response** | | | |  |  |  |
| MGST1 | Microsomal glutathione S-transferase 1 | 47.588 | 0.345 | 28 |  |  |
| CHD1L | Chromodomain helicase DNA binding protein 1-like | 13.791 | 0.264 | 14 |  |  |
| CAT | Catalase | 23.032 | 0.232 | 98 |  |  |
| PCNA | Proliferating cell nuclear antigen | 15.805 | -0.195 | 289 |  |  |
| RPA2 | Replication protein A2 | 15.621 | -0.264 | 61 |  |  |
| DUT | Deoxyuridine triphosphatase | 20.359 | -0.425 | 88 |  |  |
| SESN3 | Sestrin 3 | 3.303 | -0.552 | 4 |  |  |
| EEPD1 | Endonuclease/exonuclease/phosphatase family domain containing 1 | 5.189 | -0.599 | 6 |  |  |
| **Apoptosis** |  |  |  |  |  |  |
| BBC3 | BCL2 binding protein component 3 or PUMA | 10.918 | 0.818 | 3 |  |  |
| DAD1 | Defender against cell death 1 | 130.245 | 0.202 | 53 |  |  |
| TXNIP | Thioredoxin interacting protein | 114.451 | -0.52 | 4 |  |  |
| **Cell cycle** |  |  |  |  |  |  |
| SEPT9 | Septin 9 | 53.076 | -0.076 | 180 |  |  |
| CDK6 | Cyclin-dependent kinase 6 | 5.393 | -0.192 | 31 |  |  |
| CNNM3 | Cyclin M3 | 14.289 | -0.379 | 37 |  |  |
| GAS6 | Growth arrest-specific 6 | 19.122 | -0.743 | 9 |  |  |
| MKI67 | Marker of proliferation Ki-67 | 0.038 | -1.479 | 179 |  |  |
| **Extracellular matrix** | |  |  |  |  |  |
| P4HA2 | Prolyl 4-hydroxylase, alpha polypeptide II | 51.97 | 0.122 | 27 |  |  |
| ITGB1BP1 | Integrin beta 1 binding protein 1 | 18.391 | -0.303 | 4 |  |  |
| PXDN | Peroxidasin | 9.692 | -0.368 | 23 |  |  |
| HAPLN4 | Hyaluronan and proteoglycan link protein 4 | 9.169 | -0.582 | 27 |  |  |
| MATN2 | Matrilin 2 | 5.614 | -0.679 | 46 |  |  |
| FN1 | Fibronectin 1 | 64.511 | -0.979 | 447 |  |  |
| **Other/unknown function** | |  |  |  |  |  |
| METTL1 | Methyltransferase like 9 | 8.856 | 0.66 | 9 |  |  |
| CHPF2 | Chondroitin polymerizing factor 2 | 17.223 | 0.474 | 21 |  |  |
| TMEM135 | Transmembrane protein 135 | 11.736 | 0.457 | 4 |  |  |
| HID1 | HID-domain containing | 88.943 | 0.373 | 76 |  |  |
| MYBBP1A | MYB binding protein (P160) 1a | 21.639 | 0.356 | 307 |  |  |
| CDV3 | CDV3 homolog | 108.446 | 0.329 | 12 |  |  |
| FRY | Furry homolog (Drosophila) | 11.117 | 0.324 | 1 |  |  |
| MAP7D1 | Map 7 domain containing 1 | 60.514 | 0.311 | 10 |  |  |
| FAM114A1 | Family with sequence similarity 114, member A1 | 19.325 | 0.28 | 22 |  |  |
| MPHOSPH10 | M-phase phosphoprotein 10 | 14.527 | 0.242 | 5 |  |  |
| SUCO | SUN domain containing ossification factor | 13.696 | 0.224 | 8 |  |  |
| SLMO2 | Slowmo homolog 2 | 37.385 | 0.209 | 2 |  |  |
| ZFAND3 | Zinc finger, AN-type 1 domain 3 | 55.352 | 0.189 | 6 |  |  |
| ANKRD11 | Ankyrin repeat domain 11 | 46.507 | 0.146 | 3 |  |  |
| FAM46A | Family with sequence similarity 46, member A | 14.967 | 0.113 | 7 |  |  |
| SKP1 | S-phase kinase-associated protein 1 | 67.644 | -0.114 | 23 |  |  |
| CMIP | c-Maf inducing protein | 46.142 | -0.116 | 14 |  |  |
| TNS3 | Tensin 3 | 24.339 | -0.139 | 33 |  |  |
| RAB14 | RAB14, member RAS oncogene family | 29.969 | -0.143 | 151 |  |  |
| PPL | periplakin | 36.797 | -0.145 | 34 |  |  |
| CGGBP1 | CGG triplet repeat binding protein 1 | 23.377 | -0.148 | 17 |  |  |
| CDK5RAP2 | CDK5 regulatory subunit associated protein 2 | 11.787 | -0.171 | 4 |  |  |
| TMEM2 | transmembrane protein 2 | 19.731 | -0.176 | 56 |  |  |
| CHDH | Choline dehydrogenase | 9.92 | -0.207 | 36 |  |  |
| KANSL1 | KAT8 regulatory NSL complex subunit 1 (KIAA1267, histone acetylation) | 28.371 | -0.210 | 5 |  |  |
| ANKRD52 | Ankyrin repeat domain 52 | 12.545 | -0.213 | 29 |  |  |
| MTMR10 | Myotubularin related protein 10 | 9.397 | -0.214 | 7 |  |  |
| RNF145 | Ring finger protein 145 | 32.026 | -0.219 | 1 |  |  |
| IREB2 | Iron-responsive element binding protein 2 | 11.762 | -0.220 | 16 |  |  |
| PHLDB1 | Pleckstrin homology-like domain, family B, member 1 | 10.57 | -0.233 | 5 |  |  |
| KIAA0232 | KIAA0232 | 8.386 | -0.240 | 7 |  |  |
| DYNLL1 | Dynein, light chain, LC8-type 1 | 142.97 | -0.258 | 20 |  |  |
| DAG1 | Dystroglycan 1 (dystrophin-associated glycoprotein 1) | 29.808 | -0.263 | 6 |  |  |
| NCOA2 | Nuclear receptor coactivator 2 | 8.065 | -0.256 | 29 |  |  |
| C1ORF198 | Chromosome 1 Open Reading Frame 198 | 14.520 | -0.259 | 6 |  |  |
| LY75 | Lymphocyte antigen 75 | 4.648 | -0.28 | 1 |  |  |
| ARRB1 | Arrestin, beta 1 | 4.935 | -0.288 | 8 |  |  |
| MTSS1 | Metastasis suppressor 1 | 17.483 | -0.294 | 20 |  |  |
| FAM102A | Family with sequence similarity 102, member A | 130.881 | -0.294 | 60 |  |  |
| RRM1 | Ribonucleotide reductase M1 | 16.399 | -0.311 | 40 |  |  |
| DPY19L4 | dpy-19-like 4 (C. elegans) | 6.621 | -0.325 | 6 |  |  |
| MPHOSPH9 | M-phase phosphoprotein 9 | 5.589 | -0.342 | 3 |  |  |
| NAA40 | N(alpha)-acetyltransferase 40, NatD catalytic subunit | 12.524 | -0.335 | 12 |  |  |
| SVOP | SV2 related protein homolog (rat) | 17.571 | -0.369 | 8 |  |  |
| BAIAP2 | BAI1-associated protein 2 | 14.685 | -0.388 | 37 |  |  |
| FAM115A | TRPM8-TRPM8 channel-associated factor 1 | 20.883 | -0.395 | 4 |  |  |
| GPALPP1 | GPALPP Motifs Containing 1 | 8.405 | -0.391 | 2 |  |  |
| SNX29 | Sorting nexin 29 (RUNDC2A) | 6.28 | -0.403 | 22 |  |  |
| VASH1 | Vasohibin 1 | 11.014 | -0.431 | 10 |  |  |
| CGNL1 | Cingulin-like 1 | 5.695 | -0.422 | 1 |  |  |
| ACTR6 | ARP6 actin-related protein 6 homolog (yeast) | 5.464 | -0.504 | 12 |  |  |
| CYS1 | Cystin1 | 4.583 | -0.510 | 2 |  |  |
| SORBS2 | Sorbin and SH3 domain containing 2 | 93.872 | -0.585 | 32 |  |  |
| HRSP12 | Heat-responsive protein 12 | 4.437 | -0.582 | 27 |  |  |
| FAM171A2 | Family with sequence similarity 171, member A2 | 2.765 | -0.620 | 5 |  |  |
| PDZK1IP1 | PDZK1 interacting protein 1 | 71.804 | -0.718 | 82 |  |  |
| SLIT1 | Slit guidance ligand 1 | 2.201 | -0.868 | 39 |  |  |
| FAM179A | Family with sequence similarity 179, member A | 1.362 | -0.950 | 1 |  |  |
| NHSL2 | NHS-like 2 | 0.209 | -1.196 | 2 |  |  |
| TENM2 | Teneurin transmembrane protein 2 | 0.598 | -1.249 | 9 |  |  |

**Supplementary Table 3: siRNAs**

| **Gene** | **Species** | **Reference** | **Supplier** | **Sequence** | |
| --- | --- | --- | --- | --- | --- |
| None |  | Negative Control siRNA | Qiagen | Not provided |  |
| Creb3l2  si #1 | Rattus  norvegicus | RSS324942 | Invitrogen | CGAGGGCUAUCCCAUUCCAACCAAA  UUUGGUUGGAAUGGGAUAGCCCUCG |  |
| Creb3l2  si #2 | Rattus  norvegicus | RSS324944 | Invitrogen | UGUGCUUUGCUUUGCGGUUGCAUUU  AAAUGCAACCGCAAAGCAAAGCACA |  |
| CREB3L2 | Human | HSS127718 | Invitrogen | CCUCAUGGUUGUGGUGCUGUGCUUU  AAAGCACAGCACCACAACCAUGAGG |  |
| Bach1  si #1 | Rattus  norvegicus | RSS314826 | Invitrogen | UACUCAGCCUCAGACUGUCCGCUUU  AAAGCGGACAGUCUGAGGCUGAGUA |  |
| Bach1  si #2 | Rattus  norvegicus | SO-2565271G  On-TARGETplus SMART pool | Dharmacon | GCUACUUCCACUCGCGAAU  GCAGGAGCCUUGCCCGUAU  UUUCAAUGCUCAACGAAUA  CGUACACAAUAUCGAGGAA |  |
| BACH1 | Human | HSS100909 | Invitrogen | GGGCACCAGGGAAGAUAGUAGUGUU  AACACUACUAUCUUCCCUGGUGCCC |  |
| Slc7a5 | Rattus norvegicus | RSS328942 | Invitrogen | GCGACUAUGCCUACAUGCUAGAGGU  ACCUCUAGCAUGUAGGCAUAGUCGC |  |
| Slc7a1 | Rattus norvegicus | RSS303958 | Invitrogen | CCCGGAUAUAUUUGCUGUGAUUAUA  UAUAAUCACAGCAAAUAUAUCCGGG |  |

**Supplementary Table 4: Primer sequences**

| **Gene** | **Species** | **STD or qRT** | **Forward primer**  **Sequence 5’ to 3’** | **Reverse primer**  **Sequence 5’ to 3’** | **Amplicon length**  **(bp)** |
| --- | --- | --- | --- | --- | --- |
| Slc7a5 | Rattus norvegicus | STD | TTCCTCAAGCTCTGGATCG | ATCTGGATGAAGCCGAGCA | 269 |
|  |  | qRT | TCGCCACATACCTGCTCAA | CAGTTCACAGCCGTGAGTA | 106 |
| Slc7a1 | Rattus norvegicus | STD | CTTCATCATGGTGTCGGGA | GCATAAAAGCAGGTCGCTG | 174 |
|  |  | qRT | GCTCCATTGAGAACTGGCA | TCCCTCACCGTATTTCACG | 86 |
| Gapdh | Rattus norvegicus | STD | ATGACTCTACCCACGGCAAG | TGTGAGGGAGATGCTCAGTG | 975 |
|  |  | qRT | AGTTCAACGGCACAGTCAAG | TACTCAGCACCAGCATCACC | 118 |
| ACTB | Human | STD | AAATCTGGCACCACACCTTC | CCGATCCACACGGAGTACT | 775 |
|  |  | qRT | CTGTACGCCAACACAGTGCT | GCTCAGGAGGAGCAATGATC | 127 |
| Creb3l2 | Rattus norvegicus | STD | CTCTCAGAGAAGAGCGTGT | CCAGCACTCATATCCAGAG | 326 |
|  |  | qRT | CCAGCGATGGCTTCAATGA | GCTCTGTCTTGATGGTAGC | 87 |
| CREB3L2 | Human | STD | GAATGATCCTTTCCTCTCAGA | GAGTGACAGACGGAACGAGT | 275 |
|  |  | qRT | CCACATTACCACCAGTGAC | GGCTCTGTCTTGATGGATG | 208 |
| Bach1 | Rattus norvegicus | STD | CTCGCTTCTGTTCCTCTCTTTAG | CAGTCTTCACAGGATGCCAATA | 286 |
|  |  | qRT | TGGAGGAAGAAGTTGGGATTTAG | TCAGTGACCTGCAATGAGATG | 124 |
| BACH1 | Human | STD | AGGCTGATGGAGAGCTGAA | CCCTCTGGTCCAAAAGTGAA | 293 |
|  |  | qRT | CAGAAGAGGTGACAGTTAAAGGA | AAACTCCACACATTTGCACAC | 110 |
| STD: primers used for conventional PCR, qRT: primers used for real time qRT-PCR. | | | | | |
